# Large‐scale quantification of stomatal patterning in barley leaves overexpressing epidermal patterning factor 1 reveals differential stomatal density between the adaxial and abaxial surfaces and spatial heterogeneity that impact stomatal function

**DOI:** 10.1111/nph.70514

**Published:** 2025-08-29

**Authors:** Mengjie Fan, Keri‐Anne Moss, Pratham Jindal, Piotr Kasznicki, Philip Davey, Philippe P. Laissue, Tracy Lawson

**Affiliations:** ^1^ School of Life Sciences University of Essex Colchester CO4 3SQ UK

**Keywords:** barley (*Hordeum vulgare*), Chlorophyll autofluorescence, epidermal patterning factor, phenotyping, photosynthesis, spatial  heterogeneity, stomatal density, water use efficiency

## Abstract

- Stomatal density varies spatially over the leaf surface and between abaxial and adaxial leaf surfaces, with distribution greatly influencing plant photosynthesis and water use. However, methodological limitations have prevented quantification of spatial heterogeneity and its consequences for gaseous exchange in monocot crops.
- Here we introduce a simple and rapid method to image and quantify stomatal patterning over large (18 cm^2^) leaf areas *in situ*. We used this approach to assess spatial variation across the adaxial and abaxial surfaces in barley (*Hordeum vulgare* L.) wild‐type (WT) plants and mutants overexpressing the epidermal patterning factor 1 (EPF1).
- Analysing over a million stomata revealed significantly greater stomatal densities on the adaxial surface and towards the leaf tip in both genotypes. Overexpression of EPF1, however, differentially reduced stomatal densities on the two surfaces, while also increasing spatial variability, particularly on the abaxial surface, compared to WT.
- The uneven stomatal distribution proved crucial to separate simultaneous gas exchange measurements on the two surfaces, with impacts on both photosynthetic carbon gain and water use efficiency. Knowledge of the relationship between stomatal patterning and gaseous function is critical for the development of future crops with improved performance.

## Introduction

Stomata are microscopic structures formed by specialised guard cells on plant epidermal surfaces, which regulate the fundamental trade‐off between CO_2_ uptake for photosynthesis and water loss through transpiration. This dual function positions stomata as critical determinants of plant productivity, water use efficiency (WUE), and climate resilience (Bertolino *et al*., 2019; Lawson & Vialet‐Chabrand, 2019; Papanatsiou *et al*., 2019; Lawson & Jack, 2020; Pan *et al*., 2024). The density and distribution of stomata across leaf surfaces significantly influence gas exchange capacity, water conservation, and ultimately plant performance under varying environmental conditions (Hetherington & Woodward, 2003; Lawson & Blatt, 2014). While stomatal patterning has been extensively studied in dicotyledonous plants, monocots, which are globally important staple cereal crops, have received comparatively less attention despite their agricultural significance and distinct developmental pathways (Croxdale, 1998).

Monocot stomatal patterning differs fundamentally from that of dicots in both structure and developmental trajectory. While eudicots often (but not always) exhibit what appears as a ‘scattered’ stomatal arrangement of kidney‐shaped guard cells, grasses form parallel files of stomata with dumbbell‐shaped guard cells flanked by specialised subsidiary cells that enhance stomatal function (Raissig *et al*., 2017; Rudall *et al*., 2017). This unique four‐celled stomatal complex in grasses enables more rapid and efficient stomatal responses to environmental fluctuations (Franks & Farquhar, 2007). Importantly, monocots tend to be amphistomatous, with stomata on both adaxial and abaxial surfaces and often in different densities, which is a trait that is thought to maximise photosynthetic capacity but may also increase vulnerability to water loss (Farber *et al*., 2016; Watts *et al*., 2024; Zhen *et al*., 2025). These surface‐specific differences potentially allow differential regulation of gas exchange depending on environmental conditions, yet the functional consequences of such heterogeneity remain poorly understood in key cereal crops.

Stomatal development and patterning are regulated by a network of signaling peptides, with epidermal patterning factors (EPFs) playing a central role as negative regulators. EPF1 and EPF2 are secreted by developing stomatal lineage cells, which bind to receptor complexes including ERECTA (ER) family of leucine rich repeat receptor like kinases and the co‐receptor TOO MANY MOUTHS, which triggers intracellular signalling cascades that inhibit stomatal development in neighbouring cells (Hara *et al*., 2007, 2009; Hunt & Gray, 2009; Hunt *et al*., 2010). This mechanism enforces the ‘one‐cell spacing rule’ that prevents adjacent stomatal formation and optimises gas exchange efficiency (Hara *et al*., 2007; Dow *et al*., 2014; Sack & Buckley, 2016). Manipulating EPF expression has emerged as a promising approach to alter stomatal density (SD) with significant physiological consequences. In particular, overexpression of EPF1 in various species consistently reduces SD, enhances *WUE*, and improves drought tolerance (Hughes *et al*., 2017; Caine *et al*., 2019; Dunn *et al*., 2019) making it a promising and proven target for engineering climate resilient crops.

Spatial heterogeneity in stomatal distribution across leaf surfaces, along leaf axes, and within localised regions can significantly impact plant physiological performance (Weyers *et al*., 1997; Weyers & Lawson, 1997; Lawson & Weyers, 1999). Nonuniform stomatal patterns can create diffusion bottlenecks or enhance gas exchange efficiency depending on their specific arrangement (Dow *et al*., 2014; Buckley, 2019). For instance, clustered stomata function less efficiently than evenly spaced ones due to competition for CO₂ diffusion pathways and ionic resources (Papanatsiou *et al*., 2016). Understanding how genetic manipulations of SD, such as overexpression of EPF1, affect not just overall SD but also spatial distribution patterns is crucial for predicting the impact on whole plant performance. Yet such comprehensive spatial analyses have been challenging due to methodological limitations. Traditional methods for quantifying stomatal patterning have relied on extremely labour‐intensive approaches such as epidermal peels or impressions to be examined under microscopes, typically sampling only minuscule leaf areas (≤ 1 mm^2^). These approaches cannot capture the full extent of spatial heterogeneity across leaf surfaces, nor can they efficiently analyse the thousands of stomata needed for statistically robust conclusions. More recent attempts using handheld microscopes remain constrained by small fields of view, while automated approaches often require extensive training datasets that may not generalise well across species (Millstead *et al*., 2020; Jayakody *et al*., 2021; Gibbs & Burgess, 2024). These methodological limitations have particularly hindered detailed investigations in monocots, where the linear arrangement of stomata in files requires large‐scale imaging to accurately assess patterning (Ferguson *et al*., 2021; Xie *et al*., 2021).

Here, we introduce a simple, rapid, and nondestructive technique that enables large‐scale quantification of stomatal distribution using fluorescence microscopy of chlorophyll (Chl) autofluorescence combined with automated image processing. This approach facilitates analysis of over 1 million stomata across extensive leaf areas (> 18 cm^2^) without requiring staining, peeling, or other invasive sample preparations. We applied this methodology to investigate spatial variation in stomatal patterning across adaxial and abaxial surfaces in barley (*Hordeum vulgare*) wild‐type (WT) plants and transgenic lines overexpressing EPF1, which exhibit reduced SD.

Our present study using large‐scale image acquisition and spatial analyses revealed previously unrecognised heterogeneity in stomatal distribution between leaf surfaces *and* along the leaf axes, with distinct patterns between genotypes. We show that EPF1 overexpression not only reduced overall SD but also differentially affected the two leaf surfaces and altered spatial distribution patterns, particularly increasing variability on the abaxial surface. These spatial distribution patterns result in differential gas exchange measurements which affect both photosynthetic carbon gain and water loss. Our findings also establish direct links between genetic manipulation of stomatal development, resulting spatial patterning changes, and physiological consequences. These insights could provide innovative targets for engineering improved WUE in cereal crops facing increasingly erratic precipitation and rising temperatures in the near future.

## Materials and Methods

### Plant material growth and preparation

Transgenic barley (*H. vulgare* L.) lines overexpressing the TaEPF1 gene under the rice actin promoter control (Dunn *et al*., 2019) were grown along with azygous WT control lines. Seeds were surface‐sterilised (70% ethanol for 2 min and rinsed with sterile reverse osmosis (RO) water) and germinated in December 2023 and May 2024 under controlled conditions (20–22°C ± 0.5°C, 12 h photoperiod, 250 μmol m^−2^ s^−1^ photosynthetic photon flux density (PPFD)). Two weeks postgermination, seedlings were transplanted into 1 l pots (peat‐based compost) and grown for seven additional weeks in a CONVIRON ADAPTIS growth cabinet at the University of Essex (Colchester, UK) under the following conditions: 23°C, 65% RH, 14 h photoperiod, 600 μmol m^−2^ s^−1^ PPFD at canopy level. Plants were watered regularly with Hoogland solution. Gas exchange measurements were conducted using multiple LI‐6800 and LI‐6400xt infrared photosynthesis systems (LI‐COR Biosciences, Lincoln, NE, USA) between 08:00 h and 14:00 h on the third fully expanded leaf from the main tiller. The sampling area for the base was 40–42 mm from the base while the tip was 47–49.7 mm from the leaf tip, and sampling size was 40 mm. The measurement protocol followed a systematic approach: gas exchange measurements were initially performed on intact leaves, and the measurement was marked for subsequent analyses. For imaging measurements, leaves remained attached to the plants and were carefully mounted between two microscope slides for the imaging process.

### Gas exchange measurements

To examine how spatial variation in SD impacts dynamic gas exchange processes, we measured photosynthetic responses across anatomically distinct leaf regions (tip and base) of the third fully expanded barley leaf using a custom designed split chamber system (Wall *et al*., 2022). This chamber allowed simultaneous, independent measurement of gas fluxes from adaxial and abaxial surfaces. Mixed gas with controlled CO_2_ concentration at 400 ppm was supplied to the leaf chambers using two LI‐6400XT portable photosynthesis systems (LI‐COR Biosciences) at a fixed flow rate of 500 μmol s^−1^. Humidity was regulated using two LI‐610 dew point generators (LI‐COR Biosciences) to maintain a vapour pressure deficit of *c*. 1.2 kPa. Leaf temperature was controlled at 22°C with a circulating water bath connected to two cooling pads affixed to the leaf chamber to minimise thermal gradients across the leaf surface.

Boundary layer conductance (*g*_*b*_) for each leaf surface in the split chamber was determined using water‐saturated filter paper to simulate a leaf with infinite stomatal conductance. Under controlled conditions as described above, *g*_*b*_ was calculated as 0.582 mol m^−2^ s^−1^ per surface based on the relationship between transpiration rate and vapour pressure differential (Wall *et al*., 2022). This value was then incorporated into all subsequent conductance calculations.

Irradiance was provided to both leaf surfaces using two HelioSpectra DYNA LED lamps, with the white light channel set at a colour temperature of 5700 K. The total photon flux density incident on both leaf surfaces was measured and calibrated using a quantum sensor (LI‐250A; LI‐COR Biosciences) positioned within the leaf chamber to ensure equal and accurate light intensity readings on both leaf surfaces. Leaves were first adapted to the chamber environment under 100 μmol m^−2^ s^−1^ PPFD for 15 min to ensure acclimation. Subsequently, the light intensity was increased stepwise to 1000 μmol m^−2^ s^−1^ PPFD for a duration of 30 min. Gas exchange parameters, including net photosynthetic rate (*A*), stomatal conductance (*g*_sw_), and intercellular CO_2_ concentration (*C*_i_), were logged automatically every 10 s using the LI‐6400XT systems. Leaf temperatures of both surfaces were measured using Type‐K thermocouples (Omega Engineering, Norwalk, CT, USA), which were inserted through ports in the leaf chamber. Measurements were conducted at two positions on the leaf, one towards the base and one towards the tip, with each position measured twice.

### Image acquisition

Image acquisition was designed to be rapid and conservative to avoid photobleaching and any potential photodamage (Laissue *et al*., 2017) of the imaged leaf segment. Chl autofluorescence imaging was performed using a Nikon ECLIPSE Ti2‐E Inverted Microscope with a motorised XY and Z stage and a 25 mm field of view (Nikon Corporation, Tokyo, Japan). The microscope was equipped with a Crest X‐Light V3 spinning disk (Crestoptics S.p.A., Rome, Italy), but used in widefield fluorescence mode. The light source was an LDI‐7 Laser Diode Illuminator (89 North) with TTL triggering in a Ubob42 NIDAQ Ultimate Breakout Box to avoid illumination overhead (Kiepas *et al*., 2020).

Images were acquired at 16‐bit using a Teledyne Photometrics Kinetix camera (Tucson, AZ, USA). Image acquisition was controlled with NIS‐Elements AR v 5.42.02 (Build 1801) on an HP Z4 workstation (Intel Xeon 3.9 GHz, 128 GB DDR4 RAM, NVIDIA Quadro RTX4000 GPU). Leaf segments were excited at 470 nm and a photon flux density at the sample plane of 330 μmol m^−2^ s^−1^, with a 7 ms exposure time for each image plane. For Chl autofluorescence detection, we used a hard‐coated interference filters set (Semrock Inc., IDEX Corp., IL, USA) consisting of a chromatic reflector at 665 nm and a long‐pass emission filter at 664 nm. Imaging was performed using a CFI Plan Apochromat Lambda D 4× objective combined with a 1.5× tube lens zoom, yielding a final resolution of 1.11 μm per image pixel side.

Large area imaging was accomplished using the ‘Scan Large Image’ command in the NIS‐AR 6D module. For each leaf segment, boundaries were defined and focused using the ‘focus surface’ option to accommodate leaf curvature. Each field of view (2048 × 2048 pixels) was captured as a shallow *z*‐stack (with 5–9 focal planes) and collapsed into a maximum intensity projection to capture all fluorescence in areas where the leaf segment was not planar. Adjacent fields were stitched with 3% overlap.

### Image processing and quantification

**Fig. 1:**
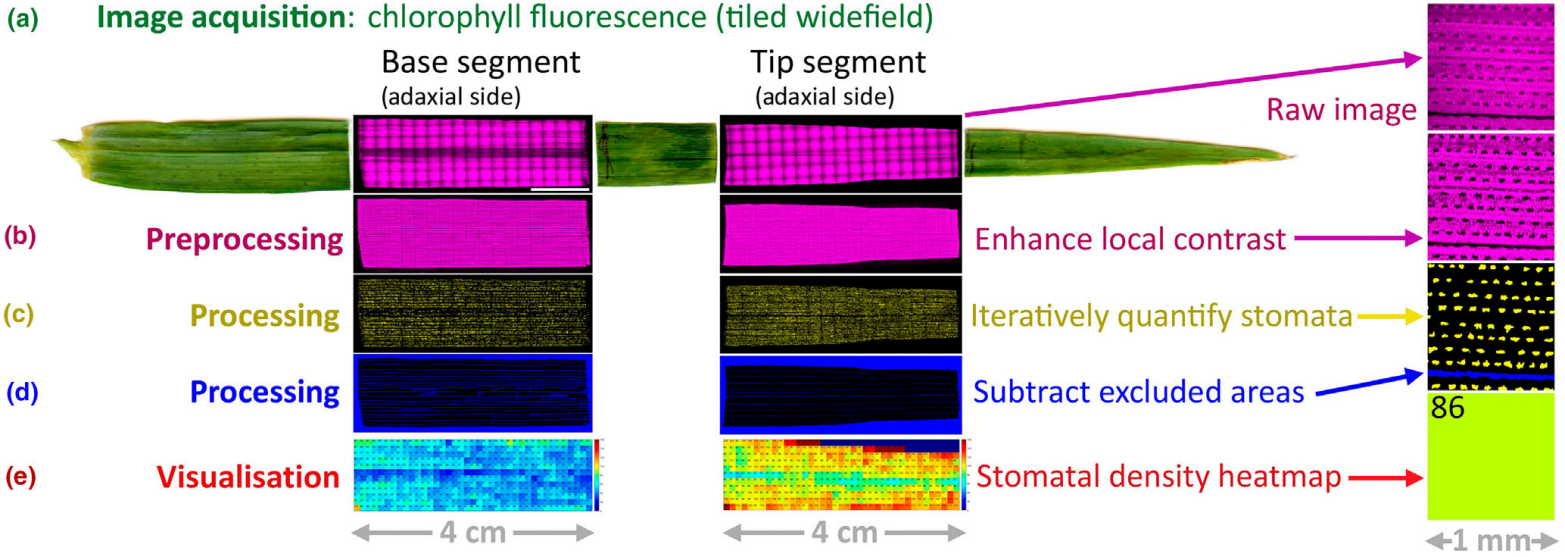
Image acquisition and image processing steps for stomatal quantification. (a) Barley flag leaf segments (at base and at tip) are imaged using tiled widefield and Chl autofluorescence. Both sides (abaxial and adaxial) of each segment are imaged. (b) Preprocessing: image contrast is enhanced locally. (c) Processing: the number and location of stomata (yellow) is determined for each side of each segment by iterative thresholding. (d) Processing: areas that cannot harbour stomata, for example leaf edge, veins or degraded areas, are determined (blue) for each side of each segment. Stomatal density is then calculated as number of stomata (yellow) per area after subtracting excluded areas (blue). (e) Visualisation: the leaf segment is divided into uniform squares of 1 mm^2^ size. The stomatal density of each is visualised using a heatmap with spectral (rainbow) colour‐coding. Numbers in heatmaps are indicated in the top left corner of each square.

Stomata were quantified using fiji v.1.54f (Schindelin *et al*., 2012). The complete workflow of image acquisition and processing is illustrated in Fig. 1. Raw images consist of large‐area (typically 38.0 ± 6.3 mm × 11.7 ± 1.4 mm), stitched Chl autofluorescence micrographs for each leaf segment, with stomata appearing as distinctive nonfluorescent elliptical regions due to the reduced chloroplast density compared with the underlying mesophyll and the thick guard cell wall that is devoid of chloroplasts (Fig. 1a).

Images were preprocessed using contrast limited adaptive histogram equalization (CLAHE) (Zuiderveld, 1994) and median filtering (Fig. 1b). For CLAHE, starting values were a block size of 127, histogram binning of 256, and contrast stretching in the intensity transfer function between 8 and 14. Depending on the available leaf (and resulting image) quality, these values may have to be modified for optimal preprocessing. The fast‐processing version of the CLAHE algorithm was used, which evaluates the intensity transfer function over the chosen block size, interpolating for locations in‐between. This was followed by a median filter of size 8. This also can be modified to optimise preprocessing for a given dataset.

Stomata were quantified using iterative thresholding of the preprocessed images. Each thresholding was followed by the ‘Analyze particles…’ command. *Moments* and *Li* thresholding had a size restriction of 800–4000 μm^2^, whereas *Minimum* used 800–3000 μm^2^. A circularity of 0.5–0.9 was used for each threshold. Areas and structures that did not harbour any stomata (such as leaf veins, borders, and degraded leaf areas) were identified using *Percentile* thresholding of areas above 400 000 μm^2^. Stomatal density was then determined as the number of stomata divided by the available area (i.e. total area minus excluded (veins, etc.) area). For the ‘heatmap’ representation, we divided each leaf into areas of 1 mm^2^. Leaf segment sizes had on average a width of 38.0 mm (±6.3 mm) and a height of 11.7 mm (±1.4 mm). The average processing time for a leaf segment, from preprocessing to stomatal quantification, was 5 min 14 s (±1 min 57 s). All raw gas exchange files and gridded stomatal matrix outputs are provided in Dataset S1.

### Calculation of global spatial variation and local spatial autocorrelation

To quantify the spatial heterogeneity in SD across the measured leaf areas, a global spatial deviation metric for each grid square within a measurement matrix was computed, where each grid square represents a 1 mm^2^ area. We calculated the absolute deviation of the SD of each focal grid square from the overall mean SD of its respective genotype, leaf position, leaf side, and plant replicate group. For a cell at row *r* and column *c* with SD_*ij*_, the absolute global deviation was computed as follows:

$$D_{\mathrm{rc}}=\left|SD_{\mathrm{rc}}-\bar{\mathrm{SD}}\right|$$

where $\bar{\mathrm{SD}}$ is the mean stomatal density calculated over the entire measurement matrix for that group. To facilitate pattern comparisons across different SD magnitudes and genotypes, the deviation for each cell was normalised by the group mean to yield a unitless, relative global deviation:

$$D_{\mathrm{rc}}^{\mathrm{rel}}=\frac{D_{\mathrm{rc}}}{\bar{\mathrm{SD}}}$$

To assess whether stomatal densities were spatially clustered or randomly distributed across the measurement grid, we computed the global Moran's *I* statistic. Using a *k*‐nearest neighbours' approach (with *k* = 4) to define spatial weights between grid squares, global Moran's *I* was calculated as follows:

$$I=\frac{n}{W}\frac{\sum_{i=1}^{n}\;\sum_{j=1}^{n}\;w_{\mathrm{ij}}\left(SD_{i}-\bar{\mathrm{SD}}\right)\left(SD_{j}-\bar{\mathrm{SD}}\right)}{\sum_{i=1}^{n}\;{\left(SD_{i}-\bar{\mathrm{SD}}\right)}^{2}}$$

where $SD_{i}$ and $SD_{j}$ represent the stomatal density values of grid squares *i* and *j*, $\bar{\mathrm{SD}}$ is the global mean stomatal density, $w_{\mathrm{ij}}$ is the spatial weight between cells *i* and *j*, *n* is the total number of grid squares, and *W* is the sum of all spatial weights $\sum_{i=1}^{n}\;\sum_{j=1}^{n}\;w_{\mathrm{ij}}$. Spatial weights ($w_{\mathrm{ij}}$) were derived using a *k*‐nearest neighbours' approach (*k = 4*) with each cell (1 mm^2^) connected to its four nearest neighbours. Positive values of Moran's *I* indicated clustering of similar stomatal density values and negative values suggested dispersion and values near zero implied spatial randomness. Moran's *I* was calculated independently for each combination of genotype, leaf position (tip and base), leaf surfaces (adaxial and abaxial), and their biological replicate to identify global spatial patterns.

To identify localised patterns and to capture spatial clustering at finer scale, we computed local Moran's *I* ($I_{i}$) for each focal grid square ($SD_{i}$):

$$I_{i}=\left(SD_{i}-\bar{\mathrm{SD}}\right)\sum_{j}\;w_{\mathrm{ij}}\left(SD_{j}-\bar{\mathrm{SD}}\right)$$

where $SD_{i}$ is the stomatal density of the grid square, $SD_{j}$ represents stomatal densities of neighbouring grid squares, $\bar{\mathrm{SD}}$ is the global mean stomatal density, and $w_{\mathrm{ij}}$ is the spatial weight between grid squres *i* and *j*. The same *k*‐nearest neighbour weights (*k = 4*) were applied. The magnitude of $I_{i}$ was interpreted as the strength of local spatial autocorrelation, whereas the sign indicated the pattern type, with positive values indicated clustering of similar values, and negative values indicated local outliers. Heatmaps of local spatial autocorrelation were reconstructed by aggregated across replicates using median values at corresponding spatial positions.

### Statistical analysis

Statistical analyses were conducted using R (v.4.4.1; R Core Team, 2024). For each variable, normality (Shapiro–Wilk test) and homogeneity of variances (Levene's test) were assessed across genotype, leaf position, and leaf surfaces groups. When both assumptions were met (*P* > 0.05), three‐way ANOVA was performed with genotype, leaf position, and leaf surfaces as fixed factors, followed by *post hoc* analysis using estimated marginal means with Sida's correction. If either assumption was violated, non‐parametric Kruskal‐Wallis tests were employed for genotype, leaf position, and leaf surfaces separately, with Dunn's test (Bonferroni‐corrected) used for *post hoc* comparisons. Data were visualised using box plots overlaying violin plots (with 95% compatibility intervals indicated by indentations) and a quasi‐random distribution of data, along with displaying effect sizes. Corresponding *P*‐values were produced using randomisation tests (Hooton, 1991; Nuzzo, 2017; Goedhart, 2019).

### Stomatal sampling simulation analysis

To evaluate optimal sampling requirements for reliable SD quantification, we implemented a Monte Carlo simulation framework using our selected SD matrix dataset (406.7 ± 74.3 mm^2^ per leaf segment). For each experimental group (genotype × leaf position × leaf side × plant replicate), we performed 1000 random sampling iterations without replacement for every possible sample size (1 to *n* total cells) using parallel processing in R (v.4.4.1). Each iteration quantified: sample mean SD, St.Dev, absolute error, relative error, SE, and 95% confidence intervals (using *t*‐distribution). Minimum sampling requirements were defined as the smallest sample size simultaneously achieving ≤ 10% mean relative error and ≥ 95% confidence interval coverage of the true population mean. To assess how sampling intensity influences structure–function relationships, we evaluated SD – *g*_sw_ correlations across three distinct sampling regimes: small (0.5–3 mm^2^, ≤ 0.5% of leaf area), medium (10–30 mm^2^, *c*. 5.1%), and large (50+ mm^2^, *c*. 34.8%) by linear regression analysis with significance testing of coefficients using Benjamini–Hochberg adjusted *P*‐values.

## Results

### Validation of the automated detection of stomata

**Fig. 2:**
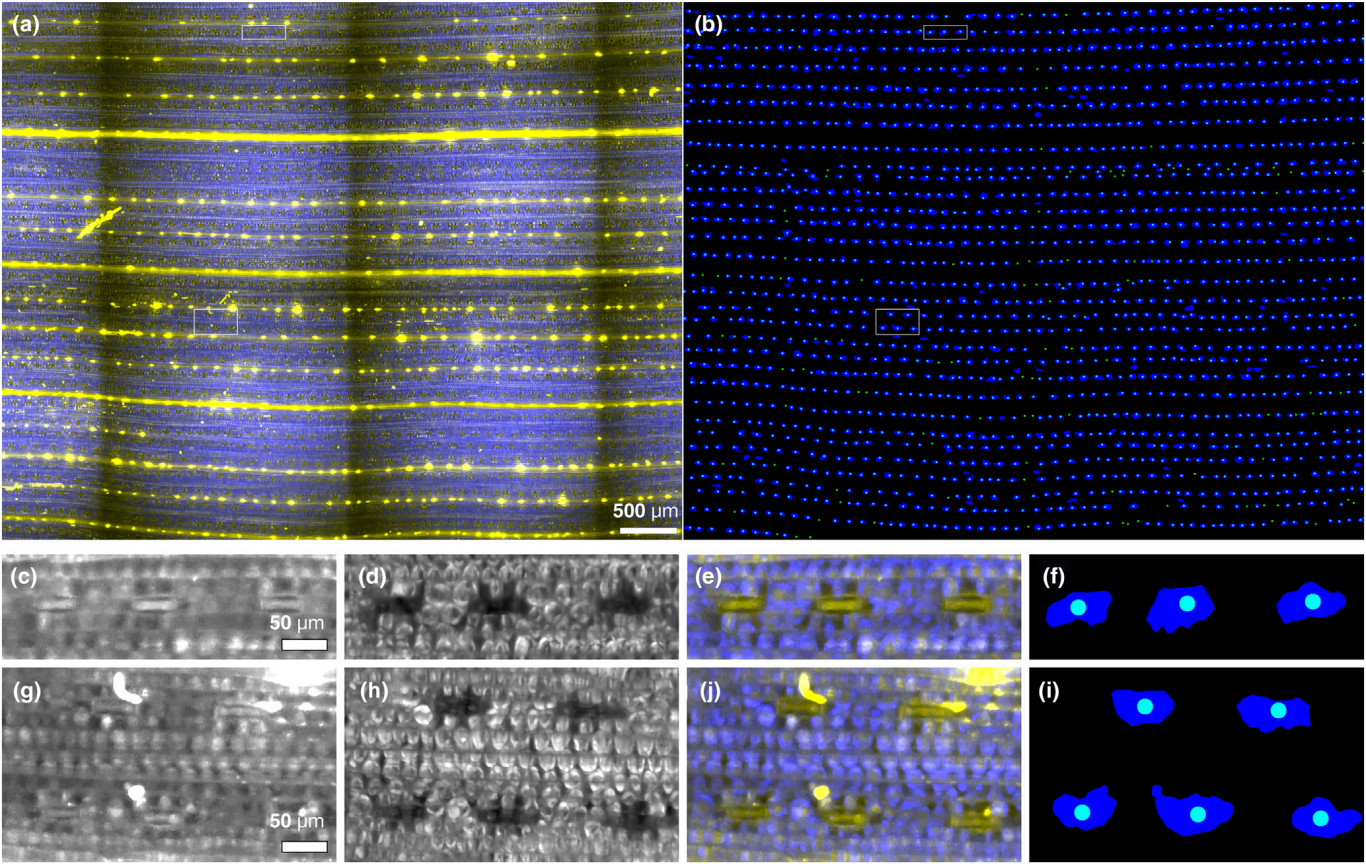
Validation of automatic stomatal detection. (a) Large image of a wild‐type leaf (abaxial tip segment, 6.0 mm × 4.7 mm) showing Chl autofluorescence (blue) with stomatal autofluorescence (yellow) overlaid. Stomatal autofluorescence further reveals leaf veins and other leaf features. (b) Manually identified stomata (green dots) overlaid with the outlines of the automatically identified stomata. The white rectangles indicate the location of the magnified insets shown in (c–f) and (g–j). Bar, 500 μm. (c–j) Magnified examples of stomatal identification. (c, g) Stomatal autofluorescence highlights the closed stomatal guard cells as a line. (d, h) Chl autofluorescence reveals stomatal locations by the absence of fluorescence. (e, j) Overlay shows that stomatal autofluorescence (yellow) colocalises with the absence of Chl autofluorescence (blue) (f, i) outline of automatically identified stomata (blue) colocalising with manually identified stomata (green dots). Bars, 50 μm. Non‐specific signals (fibres and dust particles), as seen in the stomatal autofluorescence channel, are excluded using our approach, while stomata are still reliably identified (g–j).

In order to demonstrate that our automated detection of stomata over a large area provided a good estimation of SD, values were compared with manual standard counting methods. Fig. 2 provides an example of a large autofluorescence image (Fig. 2a) adjacent to a processed image (Fig. 2b) with manual counts overlaying stomata identified using automated detection. From this large‐scale image, 1668 stomata were manually detected compared with 1619 automatically detected, demonstrating 97.1% of ‘ground truth’ stomata were identified using our automated method. Additional validation of the automated method detection is provided in Supporting Information Fig. S3, in which 10 randomly selected 1 mm^2^ squares were chosen from each leaf region, and stomata were counted both manually and using the automated method. Irrespective of the leaf area (base or tip) or which side of the leaf (abaxial or adaxial) was measured, detection rates were all > 90% (Fig. S3), with an average detection accuracy of 95.2% ± 3.6% St.Dev. providing full validation of the approach.

### Epidermal patterning factor 1 overexpression results in distinct differential spatial stomatal patterning

**Fig. 3:**
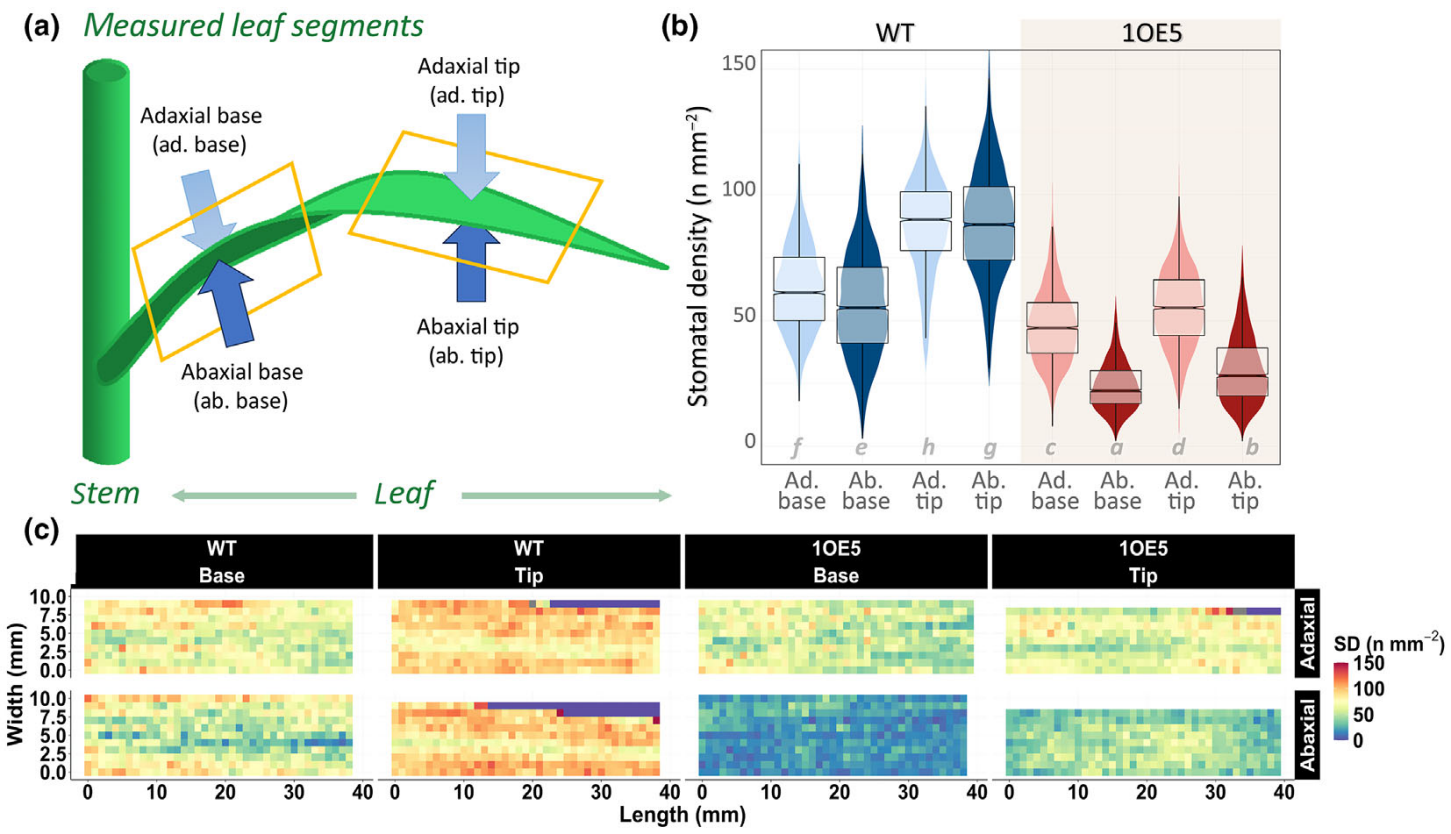
Stomatal density (SD) distribution and spatial patterns in barley. (a) Sampling layout showing base and tip segments imaged on both adaxial (Ad.) and abaxial (Ab.) leaf surfaces used in gas exchange and Chl autofluorescence imaging. (b) Box plots showing stomatal density distribution (stomata mm^−2^) in wild‐type (WT, blue colours) and EPF1 overexpressed (1OE5, red colours) barley. Ad. surfaces are light blue and light red, Ab. surfaces are dark blue and dark red. Within each box (interquartile range), the thick black line shows the median density of the segment, while indentations show the 95% compatibility interval. The data points for each box plot are underlaid in colour. (c) Representative heatmaps showing spatial distribution of SD across standardised 39 × 10 mm leaf sections. Colour scale indicates SD from 0 (blue) to 150 (red) stomata mm^−2^. EPF1, epidermal patterning factor 1.

The extensive spatial variation in stomatal patterning was quantified using our newly developed high‐throughput imaging methodology that enabled analysis of over 1 million stomata across 18 205 mm^2^ of leaf tissue and surfaces (Fig. 3a). We show the stomatal distribution across both adaxial and abaxial surfaces at two leaf positions (base and tip) and in a monocot species. WT barley exhibited substantial spatial gradients in SD, with significantly higher SD at the leaf tip compared to the base region on both leaf surfaces (Fig. 3b). The tip‐to‐base ratio was remarkably consistent between surfaces (adaxial: 1.55, abaxial: 1.54), indicating a coordinated stomatal development. Interestingly, WT maintained nearly equivalent SD between adaxial and abaxial surfaces at both leaf positions (adaxial : abaxial ratio at base: 1.03, and tip: 1.04), which is similar to that observed in wheat (Wall *et al*., 2022) but contrasting with the typical abaxial‐dominant pattern seen in many dicot species (Muir *et al*., 2014). EPF1 overexpression (1OE5) greatly altered stomatal patterning, with significant reductions in SD across all measured regions compared to WT (Fig. 3b). However, the magnitude of this effect varied substantially by both leaf position and surface. The most significant reductions can be seen on the abaxial surface, with decreases of 59.5% at the base and 64.9% at the tip. By contrast, the adaxial surface exhibited more moderate reductions of 19.9% at the base and 40.1% at the tip. This differential effect resulted in substantially altered adaxial : abaxial ratios in 1OE5 plants (base: 2.04, tip: 1.77), potentially suggesting EPF1 overexpression disproportionately impacts abaxial stomatal development. While the positional gradient in SD (in which tip > base) was preserved in 1OE5 plants, its magnitude was significantly diminished compared to WT, particularly on the adaxial surface (tip‐to‐base ratio at adaxial: 1.16, abaxial: 1.33) (Fig. 3b).

Spatial heterogeneity analysis revealed that EPF1 overexpression not only reduced mean SD but also altered the pattern of stomatal distribution across the leaf surface. Representative heat maps of SD (Fig. 3c) demonstrated that 1OE5 plants exhibited more visible spatial variability, particularly on the abaxial surface, where the coefficient of variation reached 46.2% compared to 28.7–39.0% in WT. There was no significant difference in leaf width at either the base or tip between genotypes, although as expected the tip width of each leaf was lower than its base width (Fig. S2).

### Surface‐specific gas exchange responses with overexpression of epidermal patterning factor 1

**Fig. 4:**
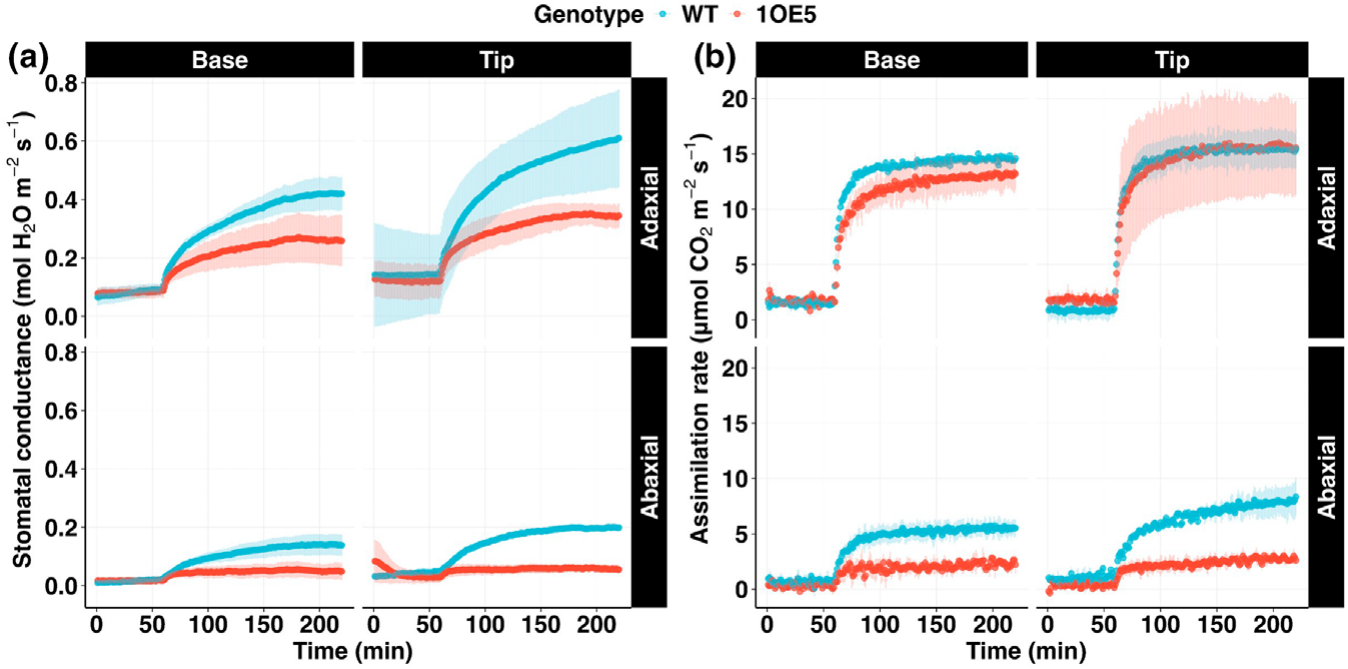
Gas exchange in wild‐type (blue). EPF1‐overexpressing (1OE5) (red) barley lines. Simultaneous and independent measurements of (a) stomatal conductance (*g*_sw_) and (b) Net CO₂ assimilation rate (*A*) on the adaxial and abaxial surfaces in response to a step increase in light intensity (from 100 to 1000 μmol m^−2^ s^−1^ PPFD). Measurements were performed at the leaf base and tip using a custom‐designed split‐chamber system. Data points represent means ± SE (*n* = 5). EPF1, epidermal patterning factor 1.

Gas exchange measurements revealed significant interactions between genotype, leaf position, and leaf surface (Fig. 4). Stomatal conductance patterns closely mirrored the photosynthetic responses, with WT maintaining consistently higher values than 1OE5 across all measured regions (Fig. 4a). The genotype effect was particularly evident at the leaf tip, where WT adaxial *g*_sw_ exceeded 1OE5 by 79% (Table S2). Notably, EPF1 overexpression had a more severe impact on abaxial conductance, with WT values being nearly three times higher than 1OE5 at the tip (0.20 vs 0.06 mol H₂O m^−2^ s^−1^) and 2.8 times higher at the base (0.14 vs 0.05 mol H₂O m^−2^ s^−1^). Under steady‐state conditions, the impact of EPF1 overexpression on photosynthesis was most pronounced in the differential response between adaxial and abaxial surfaces (Fig. 4b). In 1OE5, adaxial photosynthetic rates exceeded abaxial values by 462% at the base and 463% at the tip. By contrast, WT exhibited more moderate adaxial–abaxial differences of 163% at the base and 91% at the tip. Despite these surface differences, both genotypes achieved similar maximum photosynthetic rates on the adaxial surface at the leaf tip (*c*. 15 μmol CO₂ m^−2^ s^−1^, Fig. 4a).

### Spatial heterogeneity of stomatal distribution differs between genotypes and leaf surfaces

**Fig. 5:**
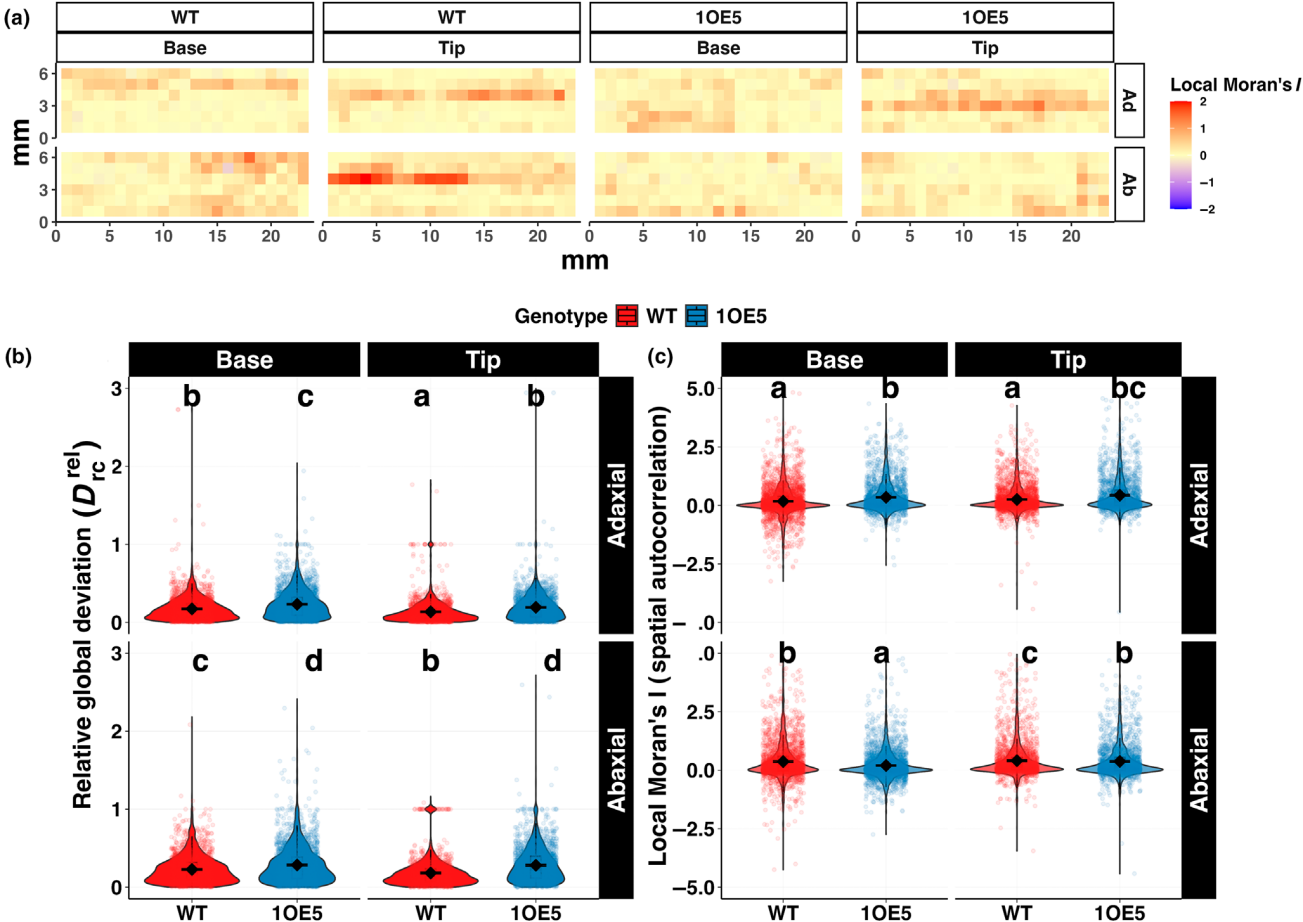
Spatial analysis of stomatal distribution patterns in wild‐type and EPF1‐overexpressing (1OE5) barley. (a) Heatmaps displaying median local Moran's *I* spatial autocorrelation values aggregated across 4–5 biological replicates at each spatial location. The colour scale ranges from red regions indicating positive spatial autocorrelation (clustering of similar values), through light yellow (zero) to, blue regions showing negative spatial autocorrelation (local outliers). Grid cells represent 1 mm^2^ areas. (b) Relative global deviation in stomatal density across genotypes (wild‐type (WT) vs 1OE5), leaf positions (base vs tip), and leaf surfaces (adaxial vs abaxial). (c) Local Moran's *I* spatial autocorrelation values (*n* = 1479–2775). Black diamonds and error bars show mean ± SE. Letters denote significant differences between groups (*P* < 0.05). EPF1, epidermal patterning factor 1.

To comprehensively analyse stomatal spatial patterning, we developed quantitative metrics assessing both global heterogeneity and local spatial autocorrelation across leaf surfaces (Fig. 5). Our approach used two complementary measures, with relative global deviation (measuring overall heterogeneity by comparing SD in each cell relative to the global mean of the whole image) and local Moran's *I* statistic (quantifying the degree of spatial clustering by assessing similarity with neighbouring regions) (Fig. 5). The relative global deviation analysis revealed significant differences in stomatal distribution patterns between WT and 1OE5 plants (*P* < 0.001), with EPF1‐overexpressing lines exhibiting substantially greater spatial heterogeneity across all measured leaf regions (Fig. 5b). This increased heterogeneity was most pronounced on the abaxial surface of the leaf base in 1OE5 plants (0.31 ± 0.003), representing a 55% increase compared to the corresponding region in WT plants (0.2 ± 0.003). Notably, we observed a significant interaction between genotype and leaf surface (*P* < 0.001), indicating that EPF1 overexpression differentially affected stomatal patterning on adaxial vs abaxial surfaces. While WT plants displayed relatively similar heterogeneity between leaf surfaces at the tip (adaxial: 0.107 ± 0.003; abaxial: 0.147 ± 0.003), 1OE5 plants showed greater disruption of spatial uniformity on the abaxial surface (adaxial: 0.178 ± 0.003; abaxial: 0.265 ± 0.003). This surface specific effect suggests distinct regulatory mechanisms controlling stomatal development on opposite leaf sides, with EPF1 disproportionately influencing the spatial coordination of stomatal initiation and differentiation more strongly on abaxial surfaces.

Local spatial autocorrelation analysis measured using Moran's *I* statistic provided additional insights into the degree to which neighbouring cells have similar stomatal densities (Fig. 5a,c). Mean local Moran's *I* values were generally low across all conditions ranging from 0.211 to 0.562, indicating predominantly random stomatal arrangements with localised regions of spatial structure. Interestingly, WT plants exhibited higher spatial autocorrelation on abaxial surfaces (tip: 0.562 ± 1.28; base: 0.385 ± 0.88) compared to adaxial surfaces (tip: 0.268 ± 0.88; base: 0.213 ± 1.09), while 1OE5 plants displayed a reversed pattern with stronger autocorrelation on adaxial surfaces (tip: 0.474 ± 1.15; base: 0.389 ± 0.88) than abaxial surfaces (tip: 0.387 ± 0.89; base: 0.211 ± 0.68). These opposing patterns between genotypes suggest that EPF1 overexpression not only alters SD but also disrupts the surface‐specific organisation. The spatial autocorrelation heatmaps (Fig. 5a) revealed averaged complex patterning across leaf regions, with distinct clusters of positive spatial autocorrelation (red) indicating areas where similar stomatal densities aggregate, and regions of negative autocorrelation (blue) representing boundary areas between different density zones. These patterns were more pronounced and spatially coherent in WT plants, while 1OE5 plants displayed more fragmented and irregular spatial organisation, particularly on abaxial surfaces.

### Functional relationships between stomatal patterning and physiological performance

**Fig. 6:**
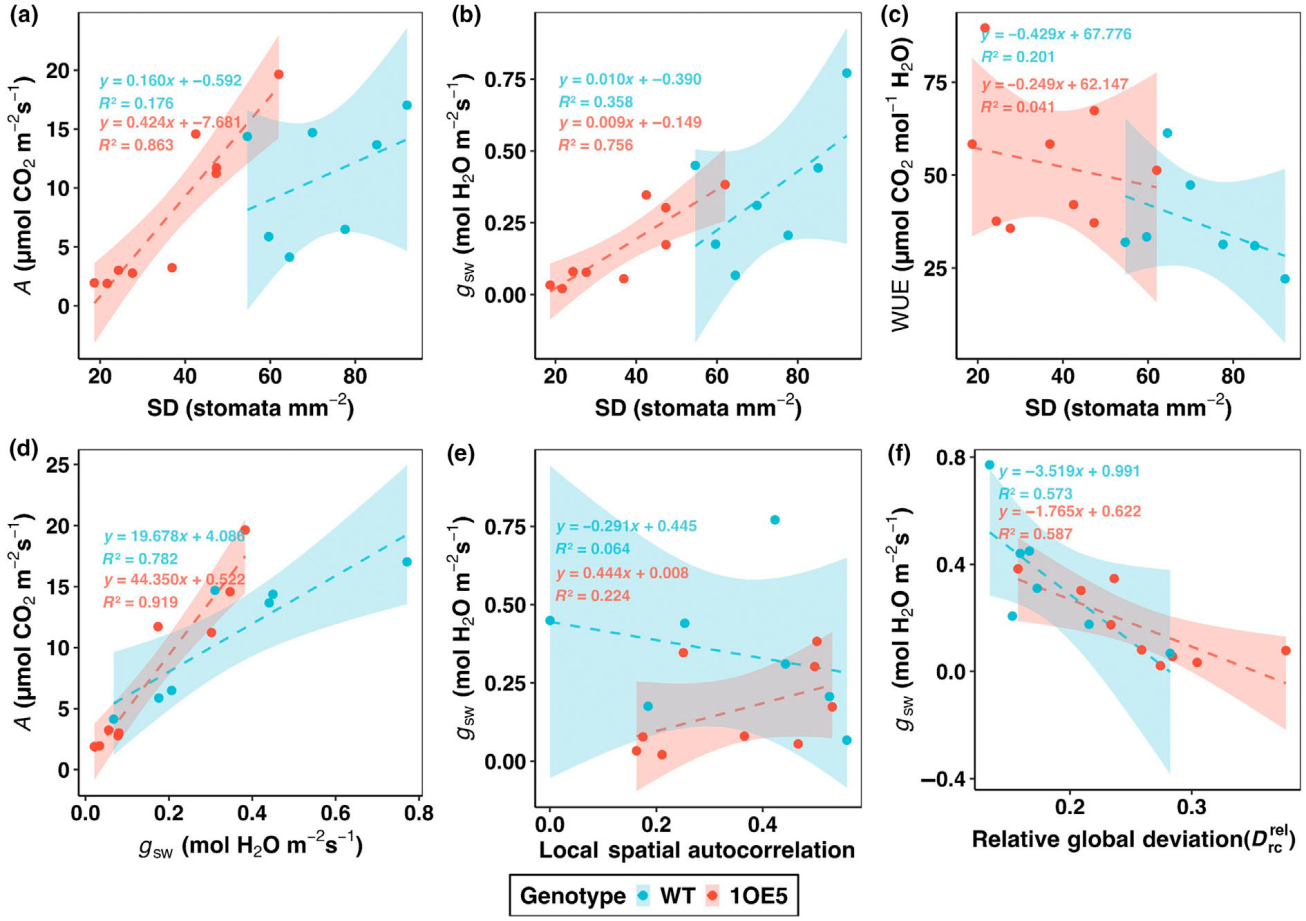
Linear regression relationships between stomatal characteristics and gas exchange parameters in wild‐type (WT) and EPF1 overexpressing (1OE5) barley. (a) Stomatal density (SD) vs net photosynthetic rate (*A*), (b) SD vs stomatal conductance (*g*_sw_), (c) SD vs water use efficiency (WUE), (d) *A* vs *g*_sw_, (e) local spatial autocorrelation vs *g*_sw_, and (f) relative global deviation vs *g*_sw_. Blue points (WT, *n* = 7) and red points (1OE5, *n* = 9) represent individual measurements with genotype‐specific regression lines and 95% confidence intervals (shaded areas). Regression equations and coefficients of determination (*R* ^2^) are provided for each genotype. EPF1, epidermal patterning factor 1.

To understand the physiological implications of altered stomatal patterning, the relationship between SD, spatial distribution metrics, and gas exchange parameters were determined (Fig. 6). As shown in Fig. 3, EPF1 overexpressing plants (1OE5) exhibited overall significantly lower SD compared to WT which corresponded with differences in physiological performance. SD showed a strong positive correlation with photosynthetic rate in 1OE5 plants (*R* ^2^ = 0.863, *P* < 0.001), but this relationship was much weaker and not significant in WT plants (*R* ^2^ = 0.176, *P* = 0.348) (Fig. 6a). The slope of this relationship was notably steeper in 1OE5 plants (0.424) compared to WT (0.160), suggesting that changes in SD had a stronger impact on photosynthetic capacity in the transgenic line. Similarly, SD was strongly correlated with *g*_sw_ in 1OE5 plants (*R* ^2^ = 0.756, *P* = 0.002), but showed only a moderate, also non‐significant relationship in WT plants (*R* ^2^ = 0.358, *P* = 0.156) (Fig. 6B). Stomatal density showed no significant correlation with WUE in either genotype (1OE5: *R* ^2^ = 0.041, *P* = 0.599; WT: *R* ^2^ = 0.201, *P* = 0.313) (Fig. 5C). Despite the lack of correlation with SD, mean WUE was 44% higher in 1OE5 plants (53.07 ± 17.75 μmol CO₂ mol^−1^ H₂O) compared to WT (36.93 ± 13.09 μmol CO₂ mol^−1^ H₂O) resulting in improved water conservation in the transgenic lines. The fundamental relationship between *A* and *g*_sw_ was strong in both genotypes (Fig. 6D), with particularly high correlation in 1OE5 plants (*R* ^2^ = 0.919, *P* < 0.001) compared to WT (*R* ^2^ = 0.782, *P* = 0.008). Notably, the steeper slope in 1OE5 (44.35) compared to WT (19.68) indicates that for a given change in *g*_sw_, *A* rates respond more sensitively in the EPF1 overexpression transgenic line, likely reflecting the reduced overall SD limiting gas exchange.

Spatial distribution metrics provided additional insights into stomatal function (Fig. 5), with no significant relationship between local spatial autocorrelation with *g*_*sw*_ in either genotype (Fig. 6e). By contrast, relative global deviation in stomatal patterning demonstrated significant negative correlations with *g*_sw_ in both 1OE5 (*R* ^2^ = 0.587, *P* = 0.016) and WT (*R* ^2^ = 0.573, *P* = 0.049) plants (Fig. 6F). This suggests that the degree of global heterogeneity in stomatal distribution, rather than local clustering patterns, is a stronger determinant of functional difference in gas exchange.

### Large‐scale stomatal sampling is essential for accurate density quantification and robust physiological correlations

**Fig. 7:**
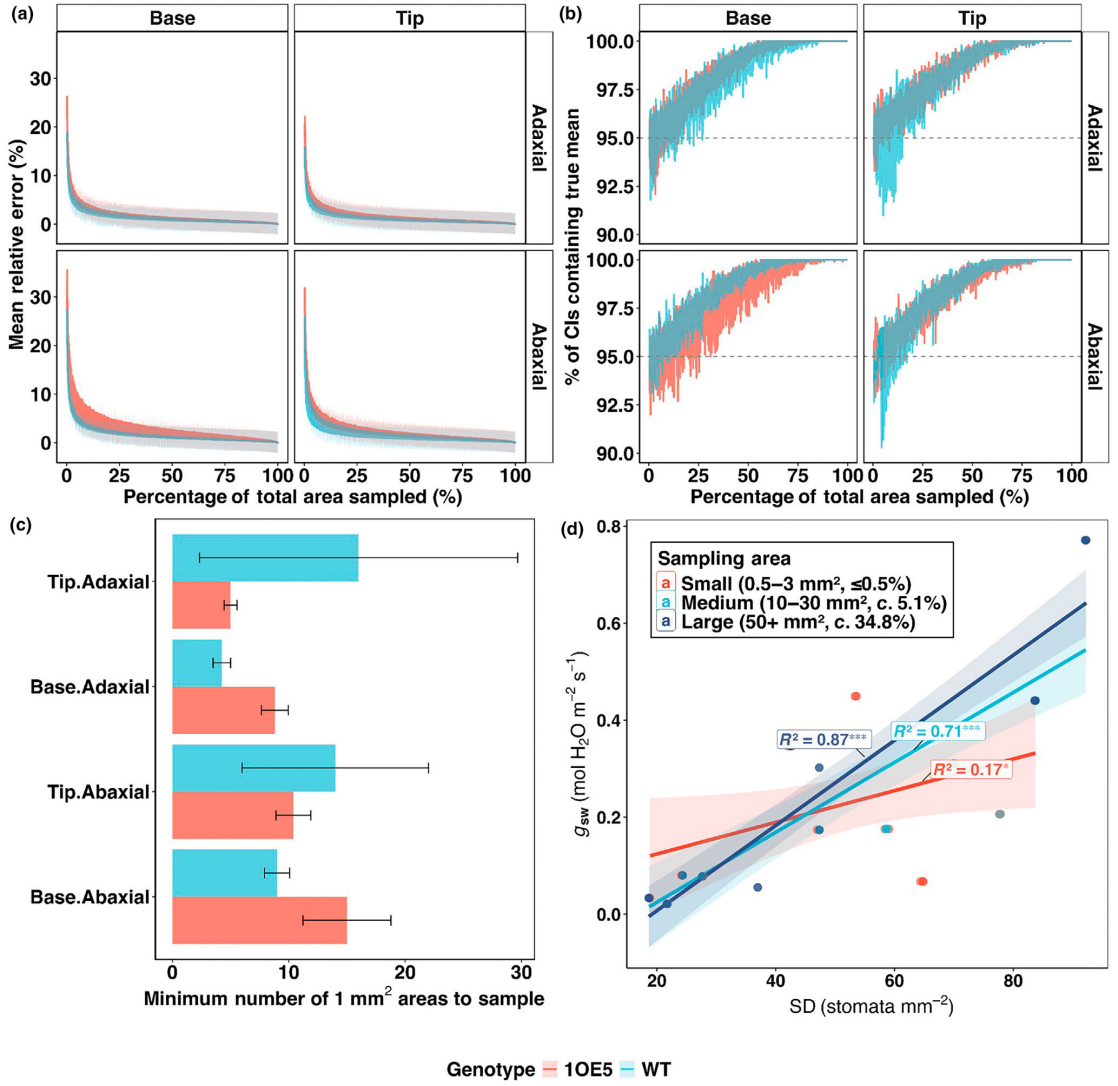
Larger‐scale stomatal sampling is essential for accurate density quantification and robust physiological correlations. (a) Mean relative error in stomatal density (SD) estimation as a function of sampling percentage in wild‐type (WT, blue) and epidermal patterning factor 1 overexpressing (1OE5, red) barley across different leaf surfaces (line types) and positions (panels). Ribbons indicate ±1 SD of mean relative error. (b) Confidence interval (CI) coverage probability (percentage of 95% CI containing true mean) as sampling percentage increases. Dashed line indicates the 95% threshold for statistical reliability. (c) Minimum sampling requirements (number of 1 mm^2^ areas) needed to achieve ≤ 10% mean relative error and ≥ 95% CI coverage across different leaf position and surface combinations. Error bars represent ±SE (*n* = 4–5 biological replicates per combination). (d) Relationship between SD and stomatal conductance as a function of sampling area size. Points represent individual measurements with sampling area‐specific regression lines and their corresponding *R* ^2^ values. Significance levels: *, *P* < 0.05; **, *P* < 0.01; ***, *P* < 0.001.

To determine the minimum sampling requirements for accurately quantifying SD across heterogeneous leaf surfaces, we conducted a comprehensive simulation analysis using our extensive matrix dataset (Fig. 7). Random sampling simulations (1000 iterations per sample size) revealed substantial variability in the area needed to obtain reliable SD estimates. While the minimum sampling requirement to achieve both acceptable accuracy (≤ 10% mean relative error) and statistical reliability (≥ 95% confidence interval coverage) ranged from as few as two 1 mm^2^ cells to as many as 57 1 mm^2^ cells (0.51–13.29% of total leaf area), our findings indicate that conventional small‐scale sampling approaches are frequently insufficient (Fig. 7a–c). The simulation revealed critical sampling thresholds varied significantly between leaf surfaces and regions. Abaxial surfaces consistently required more intensive sampling (12.2 ± 8.5 cells, 3.03% of area) compared to adaxial surfaces (8.3 ± 12.5 cells, 2.02% of area). Notably, leaf tip regions, particularly in WT plants demonstrated substantially higher sampling requirements (10.9 ± 14.1 cells, 2.87% of area) than base regions (9.6 ± 5.9 cells, 2.17% of area). The most demanding sampling conditions were observed for WT adaxial tip regions (16.0 ± 27.3 cells, 3.83% of area), where the high SD indicates that some samples required substantially more cells to achieve reliability. To eliminate sampling‐induced error across all experimental conditions, our data suggest that *c*. 57 cells (13.29% of the total leaf area) would be necessary, which far exceeds typical sampling protocols often used to determine SD.

Most critically, we demonstrated that sampling intensity fundamentally altered the strength of correlations between SD and physiological parameters (Fig. 7d). The relationship between SD and stomatal conductance (*g*_sw_) improved dramatically with increased sampling area, with *R* ^2^ values strengthening from merely 0.17 (*P* = 0.041) with small sampling areas (0.5–3 mm^2^, ≤ 0.5% of total area) to 0.71 (*P* < 0.001) with medium sampling (10–30 mm^2^, *c*. 5.1% of area) and 0.87 (*P* < 0.001) with large sampling (50+ mm^2^, *c*. 34.8% of area). This represents a 415.9% improvement in explanatory power from smallest to largest sampling, with corresponding increases in the regression slope (from 0.0033 to 0.0088). Therefore, sampling scale critically affects the reliability of stomatal to physiology relationships, with small sampling area subjects to higher probability of producing biased and inconsistent correlations.

## Discussion

Manipulating SD is a key target for altering *g*_sw_ to improve both photosynthesis and WUE (Long *et al*., 2022) and the EPF family of signalling peptides (which are negative regulators of stomatal development) are popular choices (Hara *et al*., 2007; Casson & Gray, 2008; Hunt *et al*., 2010). Increased expression of EPF1 and EPF2 has been shown to decrease SD, increase drought tolerance (Doheny‐Adams *et al*., 2012; Hepworth *et al*., 2015), as well as enhance WUE in several key crops (Hughes *et al*., 2017; Bertolino *et al*., 2019). Until recently, the majority of studies examining changes in SD have focused on changes in density, usually in a single location of the leaf lamina and on the abaxial surface, as this is often the epidermis with the greatest SD (Schlüter *et al*., 2003; Yasmeen *et al*., 2020; Bheemanahalli *et al*., 2021; Sun *et al*., 2021; Lei *et al*., 2022). Although this is frequently the case for a large number of dicotyledonous plants, monocots, including many of our major crops such as barley, have equal SD on both surfaces or even greater numbers on the adaxial surface compared with the abaxial surface (e.g. wheat, Wall *et al*., 2022). Furthermore, few studies have examined spatial variation in stomatal patterning, and even fewer have investigated how differences in SD affect gas exchange (Lawson *et al*., 1998, 2002; Lawson & Weyers, 1999) and physiological performance (Harrison *et al*., 2019; Lunn *et al*., 2024; Pflüger *et al*., 2024).

The lack of advancement in our understanding of such variation on physiological performance has been hampered by the lack of technology to assess both spatial variation in stomatal anatomical traits across large areas of the leaf, as well as methods to assess the impact on function within and between surface variation (Wall *et al*., 2022). Here we have demonstrated and validated a novel imaging method that overcomes these technological barriers which enabled comprehensive spatial analysis previously impossible with traditional sampling approaches. Traditional gas exchange approaches that use a cuvette to enclose a section of a leaf, typically measure gas exchange from both sides of the leaf simultaneously and although modifications to the kit can be made to restrict measurements to one side (Wall *et al*., 2024), most systems are not capable of measuring both leaf surfaces independently and simultaneously (Wall *et al*., 2022, 2024). Here we have demonstrated and applied a simple and novel imaging method using Chl autofluorescence to quantify over 1 million stomata across 18 cm^2^ of leaf surface area, which enabled high‐resolution spatial analysis previously impossible with traditional sampling methods.. The automated approach was validated by comparing with manual counts and demonstrated high accuracy (Figs 2, S3). This approach combining automated image processing and spatial statistical analysis revealed detailed stomatal distribution patterns that conventional sampling methods might underestimate. Although this approach facilities rapid high throughput detection of stomata it is currently limited to this single cell type, with no quantification of epidermal cell numbers or size. However, our acquired images provide the necessary spatial resolution, contrast differentiation, and standardised imaging conditions that establish an ideal foundation for comprehensive epidermal characterisation through pixel based feature classifier models. Such measurement could be extremely valuable for resolving the under lying mechanisms relating to stomatal patterning, for example, differences in spatial patterns may be due to epidermal expansion or cell differentiation (Lawson *et al*., 2002; Sack & Buckley, 2016).

Using this approach, we have demonstrated for the first time in barley (*H. vulgare*) that altered expression of the EPF1 gene differentially affects SD on the two leaf surfaces. Taking into account spatial variation across the leaf we have demonstrated that, not only does altered expression of EPF1 (Hughes *et al*., 2017) reduce SD on the abaxial epidermis more than the adaxial surface, the impact was greater at the tip than the leaf base. Several studies have demonstrated that regulation of SD using members of the EPF family have a greater effect on the abaxial epidermis (Jalakas *et al*., 2024), however, the majority of these studies have been restricted to model species such as *Arabidopsis*. Interestingly, it is not only alterations in expression of EPF genes that result in this differential patterning but disruption of other stomatal regulators have also been reported to have a greater impact on the abaxial surface (Dow *et al*., 2014; Hronková *et al*., 2015; Qi *et al*., 2019), whereas changes in ERECTA‐LIKE 2 increased adaxial but not abaxial stomatal index (Jalakas *et al*., 2024). It is currently not known if stomatal patterning is regulated by the same pathways on both surfaces. A great many studies have investigated the regulation of stomatal development and spacing and have outlined many of the signal transduction pathways, and genes involved in the process (Bergmann, 2006; Pillitteri & Dong, 2013; Wei *et al*., 2021), including the one cell spacing rule (Hara *et al*., 2007; Dow *et al*., 2014; Sack & Buckley, 2016) that ensures that stomatal initiation must be one cell away from another to ensure appropriate stomatal function (Dow *et al*., 2014; de Boer *et al*., 2016). Once again, the majority of these studies have focused on abaxial surface patterning, with much less known about stomatal development and regulation of patterning on the adaxial surface (Watts *et al*., 2024). Here the demonstration that overexpression of EPF1 has a much greater impact on SD on the abaxial surface, could be due to possible differences in the number or sensitivity of cell surface receptors on the abaxial surface, or that the EPF1 signal is not as strong at the adaxial surface. Alternatively, there are as yet unknown signalling components that influence surface specificity of stomatal development. Although there is little information available regarding the causes of differential stomatal development on the two surfaces, a recent study by Jalakas *et al*. (2024) using a range of Arabidopsis mutants in stomatal patterning supported greater effects on the abaxial surface. The authors demonstrated in mutants such as epf1/2 and er, stomatal precursors were typically found on the abaxial epidermis of fully expanded leaves. This asymmetric presence of arrested stomatal precursor cells between adaxial and abaxial surfaces further supports differential mechanisms controlling stomatal development on the two leaf surfaces.

Previous studies have also shown that the adaxial SD and index are more responsive to environmental cues such as humidity and light (Hronková *et al*., 2015; Devi & Reddy, 2018; Tulva *et al*., 2024), supporting the idea of partly independent regulation (Richardson *et al*., 2017; Wall *et al*., 2022; Jalakas *et al*., 2024). Therefore, stomatal patterning on the two surfaces may depend on both underlying genetics as well as environmental cues.

Overexpression of EPF1 not only resulted in differential differences in surface SD but also influenced spatial variation across the leaf lamina (Fig. 5), with a greater reduction at the tip than the base of the leaf. Our relative global deviation metric revealed a 55% increase in spatial heterogeneity on abaxial surfaces, with coefficients of variation reaching 46.2% in EPF1 overexpression lines compared to 28.7–39% in WT. Although significant spatial variation in SD within leaves is also well established in the literature (Smith *et al*., 1989; Poole *et al*., 2000), the majority of studies have focused on dicots, with monocots often considered to have more uniform stomatal distribution due to the ordered files (Nunes *et al*., 2020). There are only a handful of studies that have explored the impact of spatial variation in stomatal characteristics within individual leaves on leaf function and these have shown that this spatial variation along with spatial differences in microclimate result in heterogeneous patterns of leaf gas exchange (Weyers *et al*., 1997; Lawson *et al*., 1998, 2018; Lawson & Weyers, 1999; Mott *et al*., 2008; Kamakura *et al*., 2011). Interestingly in our study, SD was decreased to a greater extent at the tip than the base on both leaf surfaces. This maybe counterintuitive as it could be envisaged that the tip of the leaf is narrower due to reduced expansion of epidermal cells, which would result in higher SD (Lawson *et al*., 2002). These findings indicate that the observed variation in density is due to cell differentiation rather than expansion. An alternative plausible explanation for these observations is that the tip of the leaf is also the oldest, and therefore, it could be that older parts of the leaf signals to newly differentiating cells, as has been shown between mature and young leaves (Lake *et al*., 2001), or that as the leaf grows it becomes less sensitive to genetic regulation of SD, and/or that environment signals start to override genetic intervention.

As SD is a major determinant of functional behaviour, it is not surprising that larger decreases in *g*_sw_ were observed in the 1OE5 mutants (Fig. 4), and that the magnitude of the decrease was strongly correlated with the decrease in numbers (Fig. 6). The stronger SD and *g*_sw_ relationship observed in the 1OE5 mutants compared with WT (Fig. 6), clearly demonstrates that stomatal numbers were not optimal in the WT and that density was actually higher than required for steady‐state photosynthetic function. This is further exemplified by the fact that no significant differences in photosynthetic rates (*A*) were observed in the mutants on the adaxial surface, irrespective of leaf position (Fig. 4b). Overinvestment in stomatal and nonoptimal patterning has been reported recently by Watts *et al*. (2024), in which the authors explored patterning of stomata on both leaf surfaces under different environmental conditions and concluded that stomata are not ideally dispersed on the two surfaces, and that patterning is surface independent as well as that any coordination between the patterning on the abaxial and adaxial would result in limited improvement in photosynthesis. The greater *g*_sw_ than required for *A* in the WT results in an erosion of WUE (Lawson & Blatt, 2014; Franks *et al*., 2015). On the other hand, the substantially reduced abaxial *g*_sw_ in the mutants caused diffusional limitation on *A* (Vialet‐Chabrand *et al*., 2021), highlighting the close coupling between *A* and *g*_sw_ (Wong *et al*., 1979) at least on this surface. The greater decrease in WUE at the tip demonstrates the importance of taking into consideration spatial variation in gas exchange and WUE, as cumulatively these differences could have significant impacts on whole plant water status (Martin & Stabler, 2002; Medranoa *et al*., 2015; Brillante *et al*., 2017; Liu *et al*., 2018), with important agricultural relevance. This within leaf surface variation also highlights the importance of adequate sampling protocols. As demonstrated in our simulation analysis that conventional 1 mm^2^ samples substantially underestimate spatial heterogeneity and structural functional correlations (Fig. 7a–c). The simulation of 14.6 million sampling iterations showed that minimum sampling requirements range from 8 to 10 mm^2^ to achieve both acceptable statistical reliability and accuracy. The local Moran's *I* analysis (Fig. 5a,c) also revealed distinct clustering patterns characterised by varying degrees of spatial autocorrelation. It is worth pointing out that this spatial organisation has direct functional consequences, with relative global deviation showing significant negative correlations with stomatal conductance, revealing that uniform stomatal distribution enhances overall leaf‐level gas exchange efficiency (Fig. 6f). These spatial patterns align with developmental mechanisms governing surface specific stomatal initiation. Our findings that EPF1 overexpression increases spatial heterogeneity (relative global deviation increased by 55% on abaxial surfaces) may reflect disrupted cell–cell signalling cascades that normally enforce ordered stomatal spacing, particularly in regions where stomatal precursors preferentially accumulate in the abaxial epidermis as shown recently by Jalakas *et al*. (2024).

To our knowledge, this is the first study that reported differential effects of EPF1 expression on SD on the abaxial and adaxial leaf surface *and* measured the impacts on gaseous exchange of the two surfaces simultaneously. These findings highlight that stomatal patterning in monocots is differentially controlled in the adaxial and abaxial surfaces and raises questions regarding the signalling pathway or control of patterning on the adaxial surfaces. Furthermore, using a novel method, we have shown that altered expression of EPF1 results in SD disparities over the leaf lamina, with consequences for both *A* and water use. Such spatial variation must be considered when evaluating SD in the context of genetic modifications or physiological performance, as it can significantly influence whole‐plant water relations and affect the reliability of sampling protocols. Although we have also demonstrated an over investment in stomata on the adaxial surface, it is clear that the adaxial and not the abaxial surface supports greater gaseous diffusion and high photosynthetic rates similar to wheat (Wall *et al*., 2022) despite equal SD on both surfaces. Understanding the underlying control of stomatal patterning on both surfaces relative to photosynthetic capacity will deliver new avenues for manipulating surfaces independently providing novel targets for producing plants with optimal gaseous exchange for photosynthetic performance, evaporative cooling, and/or WUE essential, for future crop resilience given the predicted changes to climate.

## Competing interests

None declared.

## Author contributions

MF, PPL, and TL designed the experiments and wrote the manuscript. MF executed the experiments and acquired the data. PK, KAM, PJ, and PD supported data collection on the microscope and gas exchange measurements. MF and PPL developed all quantification methods and analysed all data. TL and PPL contributed equally to this work.

## Disclaimer

The New Phytologist Foundation remains neutral with regard to jurisdictional claims in maps and in any institutional affiliations.

## Supporting information


**Dataset S1** Raw gas exchange data and image matrix data.

**Fig. S1** Representative growth light spectrum measured at flag leaf level.
**Fig. S2** Leaf width variation between genotypes and positions along the barley leaf.
**Fig. S3** Manual validation of automated stomatal detection across 1 mm^2^ sampling areas.
**Table S1** Stomatal density and effect of EPF1 overexpression.
**Table S2** Coefficient of variation (%) in stomatal density.
**Table S3** Stomatal distribution ratios.
Please note: Wiley is not responsible for the content or functionality of any Supporting Information supplied by the authors. Any queries (other than missing material) should be directed to the *New Phytologist* Central Office.

## Data Availability

All raw gas exchange data and gridded stomatal matrix outputs are available in Dataset S1. Addtional materials are provided as Figs S1–S3 and Tables S1–S3 in the Supporting Information.

## References

[nph70514-bib-0001] Bergmann D . 2006. Stomatal development: from neighborly to global communication. Current Opinion in Plant Biology 9: 478–483. 16890476 10.1016/j.pbi.2006.07.001

[nph70514-bib-0002] Bertolino L , Caine R , Gray J . 2019. Impact of stomatal density and morphology on water‐use efficiency in a changing world. Frontiers in Plant Science 10: 458. 30894867 10.3389/fpls.2019.00225 PMC6414756

[nph70514-bib-0003] Bheemanahalli R , Wang C , Bashir E , Chiluwal A , Pokharel M , Perumal R , Moghimi N , Ostmeyer T , Caragea D , Jagadish S . 2021. Classical phenotyping and deep learning concur on genetic control of stomatal density and area in sorghum. Plant Physiology 186: 1562–1579. 33856488 10.1093/plphys/kiab174 PMC8260133

[nph70514-bib-0004] de Boer HJ , Price CA , Wagner‐Cremer F , Dekker SC , Franks PJ , Veneklaas EJ . 2016. Optimal allocation of leaf epidermal area for gas exchange. New Phytologist 210: 1219–1228. 26991124 10.1111/nph.13929 PMC5069575

[nph70514-bib-0005] Brillante L , Martínez‐Luscher J , Yu R , Plank C , Sanchez L , Bates T , Brenneman C , Oberholster A , Kurtural S . 2017. Assessing spatial variability of grape skin flavonoids at the vineyard scale based on plant water status mapping. Journal of Agricultural and Food Chemistry 65: 5255–5265. 28602091 10.1021/acs.jafc.7b01749

[nph70514-bib-0006] Buckley TN . 2019. How do stomata respond to water status? New Phytologist 224: 21–36. 31069803 10.1111/nph.15899

[nph70514-bib-0007] Caine RS , Yin X , Sloan J , Harrison EL , Mohammed U , Fulton T , Biswal AK , Dionora J , Chater CC , Coe RA . 2019. Rice with reduced stomatal density conserves water and has improved drought tolerance under future climate conditions. New Phytologist 221: 371–384. 30043395 10.1111/nph.15344 PMC6492113

[nph70514-bib-0008] Casson S , Gray JE . 2008. Influence of environmental factors on stomatal development. New Phytologist 178: 9–23. 18266617 10.1111/j.1469-8137.2007.02351.x

[nph70514-bib-0009] Croxdale J . 1998. Stomatal patterning in monocotyledons: tradescantia as a model system. Journal of Experimental Botany 49: 279–292.

[nph70514-bib-0010] Devi MJ , Reddy VR . 2018. Transpiration response of cotton to vapor pressure deficit and its relationship with stomatal traits. Frontiers in Plant Science 9: 1572. 30420866 10.3389/fpls.2018.01572 PMC6218332

[nph70514-bib-0011] Doheny‐Adams T , Hunt L , Franks PJ , Beerling DJ , Gray JE . 2012. Genetic manipulation of stomatal density influences stomatal size, plant growth and tolerance to restricted water supply across a growth carbon dioxide gradient. Philosophical Transactions of the Royal Society, B: Biological Sciences 367: 547–555. 10.1098/rstb.2011.0272 PMC3248714 22232766

[nph70514-bib-0012] Dow GJ , Bergmann DC , Berry JA . 2014. An integrated model of stomatal development and leaf physiology. New Phytologist 201: 1218–1226. 24251982 10.1111/nph.12608

[nph70514-bib-0013] Dunn J , Hunt L , Afsharinafar M , Meselmani MA , Mitchell A , Howells R , Wallington E , Fleming AJ , Gray JE . 2019. Reduced stomatal density in bread wheat leads to increased water‐use efficiency. Journal of Experimental Botany 70: 4737–4748. 31172183 10.1093/jxb/erz248 PMC6760291

[nph70514-bib-0014] Farber M , Attia Z , Weiss D . 2016. Cytokinin activity increases stomatal density and transpiration rate in tomato. Journal of Experimental Botany 67: erw398. 10.1093/jxb/erw398 PMC5181579 27811005

[nph70514-bib-0015] Ferguson JN , Fernandes SB , Monier B , Miller ND , Allen D , Dmitrieva A , Schmuker P , Lozano R , Valluru R , Buckler ES . 2021. Machine learning‐enabled phenotyping for GWAS and TWAS of WUE traits in 869 field‐grown sorghum accessions. Plant Physiology 187: 1481–1500. 34618065 10.1093/plphys/kiab346 PMC9040483

[nph70514-bib-0016] Franks PJ , Doheny‐Adams TW , Britton‐Harper ZJ , Gray JE . 2015. Increasing water‐use efficiency directly through genetic manipulation of stomatal density. New Phytologist 207: 188–195. 25754246 10.1111/nph.13347

[nph70514-bib-0017] Franks PJ , Farquhar GD . 2007. The mechanical diversity of stomata and its significance in gas‐exchange control. Plant Physiology 143: 78–87. 17114276 10.1104/pp.106.089367 PMC1761988

[nph70514-bib-0018] Gibbs JA , Burgess AJ . 2024. Application of deep learning for the analysis of stomata: a review of current methods and future directions. Journal of Experimental Botany 75: erae207. 10.1093/jxb/erae207 PMC11565211 38716775

[nph70514-bib-0019] Goedhart J . 2019. Plots of differences–a web app for the quantitative comparison of unpaired data. *bioRxiv* . doi: 10.1101/578575.

[nph70514-bib-0020] Hara K , Kajita R , Torii K , Bergmann D , Kakimoto T . 2007. The secretory peptide gene EPF1 enforces the stomatal one‐cell‐spacing rule. Genes & Development 21: 1720–1725. 17639078 10.1101/gad.1550707 PMC1920166

[nph70514-bib-0021] Hara K , Yokoo T , Kajita R , Onishi T , Yahata S , Peterson KM , Torii KU , Kakimoto T . 2009. Epidermal cell density is autoregulated via a secretory peptide, EPIDERMAL PATTERNING FACTOR 2 in Arabidopsis leaves. Plant and Cell Physiology 50: 1019–1031. 19435754 10.1093/pcp/pcp068

[nph70514-bib-0022] Harrison E , Cubas LA , Gray J , Hepworth C . 2019. The influence of stomatal morphology and distribution on photosynthetic gas exchange. The Plant Journal 101: 768–779. 31583771 10.1111/tpj.14560 PMC7065165

[nph70514-bib-0023] Hepworth C , Doheny‐Adams T , Hunt L , Cameron DD , Gray JE . 2015. Manipulating stomatal density enhances drought tolerance without deleterious effect on nutrient uptake. New Phytologist 208: 336–341. 26268722 10.1111/nph.13598 PMC4973681

[nph70514-bib-0024] Hetherington AM , Woodward FI . 2003. The role of stomata in sensing and driving environmental change. Nature 424: 901–908. 12931178 10.1038/nature01843

[nph70514-bib-0025] Hooton JW . 1991. Randomization tests: statistics for experimenters. Computer Methods and Programs in Biomedicine 35: 43–51. 1879135 10.1016/0169-2607(91)90103-z

[nph70514-bib-0026] Hronková M , Wiesnerová D , Šimková M , Skůpa P , Dewitte W , Vráblová M , Zažímalová E , Šantrůček J . 2015. Light‐induced STOMAGEN‐mediated stomatal development in Arabidopsis leaves. Journal of Experimental Botany 66: 4621–4630. 26002974 10.1093/jxb/erv233

[nph70514-bib-0027] Hughes J , Hepworth C , Dutton C , Dunn JA , Hunt L , Stephens J , Waugh R , Cameron DD , Gray JE . 2017. Reducing stomatal density in barley improves drought tolerance without impacting on yield. Plant Physiology 174: 776–787. 28461401 10.1104/pp.16.01844 PMC5462017

[nph70514-bib-0028] Hunt L , Bailey KJ , Gray JE . 2010. The signalling peptide EPFL9 is a positive regulator of stomatal development. New Phytologist 186: 609–614. 20149115 10.1111/j.1469-8137.2010.03200.x

[nph70514-bib-0029] Hunt L , Gray JE . 2009. The signaling peptide EPF2 controls asymmetric cell divisions during stomatal development. Current Biology 19: 864–869. 19398336 10.1016/j.cub.2009.03.069

[nph70514-bib-0030] Jalakas P , Tulva I , Berzina NM , Horak H . 2024. Stomatal patterning is differently regulated in adaxial and abaxial epidermis in Arabidopsis. *bioRxiv* . doi: 10.1101/2024.02.22.581564. PMC11523041 39158985

[nph70514-bib-0031] Jayakody H , Petrie P , Boer HJ , Whitty M . 2021. A generalised approach for high‐throughput instance segmentation of stomata in microscope images. Plant Methods 17: 1–13. 33750422 10.1186/s13007-021-00727-4 PMC7945362

[nph70514-bib-0032] Kamakura M , Kosugi Y , Takanashi S , Matsumoto K , Okumura M , Philip E . 2011. Patchy stomatal behavior during midday depression of leaf CO₂ exchange in tropical trees. Tree Physiology 31: 160–168. 21383025 10.1093/treephys/tpq102

[nph70514-bib-0033] Kiepas A , Voorand E , Mubaid F , Siegel PM , Brown CM . 2020. Optimizing live‐cell fluorescence imaging conditions to minimize phototoxicity. Journal of Cell Science 133: jcs242834. 31988150 10.1242/jcs.242834

[nph70514-bib-0034] Laissue PP , Alghamdi RA , Tomancak P , Reynaud EG , Shroff H . 2017. Assessing phototoxicity in live fluorescence imaging. Nature Methods 14: 657–661. 28661494 10.1038/nmeth.4344

[nph70514-bib-0035] Lake J , Quick W , Beerling DJ , Woodward FI . 2001. Signals from mature to new leaves. Nature 411: 154. 11346781 10.1038/35075660

[nph70514-bib-0036] Lawson T , Blatt M . 2014. Stomatal size, speed, and responsiveness impact on photosynthesis and water use efficiency. Plant Physiology 164: 1556–1570. 24578506 10.1104/pp.114.237107 PMC3982722

[nph70514-bib-0037] Lawson T , Craigon J , Black CR , Colls JJ , Landon G , Weyers JD . 2002. Impact of elevated CO_2_ and O_3_ on gas exchange parameters and epidermal characteristics in potato (*Solanum tuberosum* L.). Journal of Experimental Botany 53: 737–746. 11886894 10.1093/jexbot/53.369.737

[nph70514-bib-0038] Lawson T , Jack SAM . 2020. Guard cell metabolism and stomatal function. Annual Review of Plant Biology 20: 458. 10.1146/annurev-arplant-050718-100251 32155341

[nph70514-bib-0039] Lawson T , Terashima I , Fujita T , Wang Y . 2018. Coordination between photosynthesis and stomatal behavior. In: The leaf: a platform for performing photosynthesis. Cham, Switzerland: Springer, 141–161.

[nph70514-bib-0040] Lawson T , Vialet‐Chabrand S . 2019. Speedy stomata, photosynthesis and plant water use efficiency. New Phytologist 221: 93–98. 29987878 10.1111/nph.15330

[nph70514-bib-0041] Lawson T , Weyers J . 1999. Spatial and temporal variation in gas exchange over the lower surface of *Phaseolus vulgaris* L. primary leaves. Journal of Experimental Botany 50: 1381–1391.

[nph70514-bib-0042] Lawson T , Weyers J , A'Brook R . 1998. The nature of heterogeneity in the stomatal behaviour of *Phaseolus vulgaris* L. primary leaves. Journal of Experimental Botany 49: 1387–1395.

[nph70514-bib-0043] Lei Z , He Y , Li X , He Z , Zhang Y , Zhang W , Liu F , Zhang Y . 2022. Domestication reduces leaf water use efficiency associated with the abaxial stomatal anatomy in cotton. Journal of Experimental Botany 58: 4598. 10.1093/jxb/erac447 36385641

[nph70514-bib-0044] Liu C , He N , Zhang J , Li Y , Wang Q , Sack L , Yu G . 2018. Variation of stomatal traits from cold‐temperate to tropical forests and association with water use efficiency. Functional Ecology 32: 20–28.

[nph70514-bib-0045] Long SP , Taylor SH , Burgess SJ , Carmo‐Silva E , Lawson T , De Souza AP , Leonelli L , Wang Y . 2022. Into the shadows and back into sunlight: photosynthesis in fluctuating light. Annual Review of Plant Biology 73: 617–648. 10.1146/annurev-arplant-070221-024745 35595290

[nph70514-bib-0046] Lunn D , Kannan B , Germon A , Leverett A , Clemente T , Altpeter F , Leakey A . 2024. Greater aperture counteracts effects of reduced stomatal density on water use efficiency: a case study on sugarcane and meta‐analysis. Journal of Experimental Botany 75: 6837–6849. 39021256 10.1093/jxb/erae271 PMC11565199

[nph70514-bib-0047] Martin C , Stabler L . 2002. Plant gas exchange and water status in urban desert landscapes. Journal of Arid Environments 51: 235–254.

[nph70514-bib-0048] Medranoa H , Tomása M , Martorella S , Flexasa J , Hernándeza E , Rossellóa J , Poub A , Escalonaa J‐M , Botaa J . 2015. From leaf to whole‐plant water use efficiency (WUE) in complex canopies: limitations of leaf WUE as a selection target. Crop Journal 3: 220–228.

[nph70514-bib-0049] Millstead L , Jayakody H , Patel H , Kaura V , Petrie PR , Tomasetig F , Whitty M . 2020. Accelerating automated stomata analysis through simplified sample collection and imaging techniques. Frontiers in Plant Science 11: 580389. 33101348 10.3389/fpls.2020.580389 PMC7546325

[nph70514-bib-0050] Mott KA , Sibbernsen ED , Shope JC . 2008. The role of the mesophyll in stomatal responses to light and CO_2_ . Plant, Cell & Environment 31: 1299–1306. 10.1111/j.1365-3040.2008.01845.x 18541006

[nph70514-bib-0051] Muir CD , Hangarter RP , Moyle LC , Davis PA . 2014. Morphological and anatomical determinants of mesophyll conductance in wild relatives of tomato (Solanum sect. Lycopersicon, sect. Lycopersicoides; Solanaceae). Plant, Cell & Environment 37: 1415–1426. 10.1111/pce.12245 24279358

[nph70514-bib-0052] Nunes TD , Zhang D , Raissig MT . 2020. Form, development and function of grass stomata. The Plant Journal 101: 780–799. 31571301 10.1111/tpj.14552

[nph70514-bib-0053] Nuzzo RL . 2017. Randomization test: an alternative analysis for the difference of two means. PM & R: The Journal of Injury, Function, and Rehabilitation 9: 306–310. 10.1016/j.pmrj.2017.02.001 28237692

[nph70514-bib-0054] Pan S , Wang X , Yan Z , Wu J , Guo L , Peng Z , Wu Y , Li J , Wang B , Su Y *et al*. 2024. Leaf stomatal configuration and photosynthetic traits jointly affect leaf water use efficiency in forests along climate gradients. New Phytologist 244: 1250–1262. 39223910 10.1111/nph.20100

[nph70514-bib-0055] Papanatsiou M , Amtmann A , Blatt MR . 2016. Stomatal spacing safeguards stomatal dynamics by facilitating guard cell ion transport independent of the epidermal solute reservoir. Plant Physiology 172: 254–263. 27406168 10.1104/pp.16.00850 PMC5074606

[nph70514-bib-0056] Papanatsiou M , Petersen J , Henderson L , Wang Y , Wang Y , Christie J , Blatt M , Blatt M . 2019. Optogenetic manipulation of stomatal kinetics improves carbon assimilation, water use, and growth. Science 363: 1456–1459. 30923223 10.1126/science.aaw0046

[nph70514-bib-0057] Pflüger T , Jensen S , Liu F , Rosenqvist E . 2024. Leaf gas exchange responses to combined heat and drought stress in wheat genotypes with varied stomatal density. Environmental and Experimental Botany 228: 457.

[nph70514-bib-0058] Pillitteri LJ , Dong J . 2013. Stomatal development in Arabidopsis. The Arabidopsis Book/American Society of Plant Biologists 11: 789. 10.1199/tab.0162 PMC3711358 23864836

[nph70514-bib-0059] Poole I , Lawson T , Weyers J , Raven J . 2000. Effect of elevated CO2 on the stomatal distribution and leaf physiology of *Alnus glutinosa* . New Phytologist 145: 511–521. 33862906 10.1046/j.1469-8137.2000.00589.x

[nph70514-bib-0060] Qi S , Lin Q , Feng X , Han H , Liu J , Zhang L , Wu S , Le J , Blumwald E , Hua X . 2019. IDD16 negatively regulates stomatal initiation via *trans*‐repression of SPCH in Arabidopsis. Plant Biotechnology Journal 17: 1446–1457. 30623555 10.1111/pbi.13070 PMC6576023

[nph70514-bib-0082] R Core Team . 2024. R: A language and environment for statistical computing. Vienna, Austria: R Foundation for Statistical Computing.

[nph70514-bib-0061] Raissig MT , Matos JL , Anleu Gil MX , Kornfeld A , Bettadapur A , Abrash E , Allison HR , Badgley G , Vogel JP , Berry JA . 2017. Mobile MUTE specifies subsidiary cells to build physiologically improved grass stomata. Science 355: 1215–1218. 28302860 10.1126/science.aal3254

[nph70514-bib-0062] Richardson F , Brodribb TJ , Jordan GJ . 2017. Amphistomatic leaf surfaces independently regulate gas exchange in response to variations in evaporative demand. Tree Physiology 37: 869–878. 28898992 10.1093/treephys/tpx073

[nph70514-bib-0063] Rudall PJ , Chen ED , Cullen E . 2017. Evolution and development of monocot stomata. American Journal of Botany 104: 1122–1141. 28794059 10.3732/ajb.1700086

[nph70514-bib-0064] Sack L , Buckley TN . 2016. The developmental basis of stomatal density and flux. Plant Physiology 171: 2358–2363. 27268500 10.1104/pp.16.00476 PMC4972277

[nph70514-bib-0065] Schindelin J , Arganda‐Carreras I , Frise E , Kaynig V , Longair M , Pietzsch T , Preibisch S , Rueden C , Saalfeld S , Schmid B . 2012. fiji: an open‐source platform for biological‐image analysis. Nature Methods 9: 676–682. 22743772 10.1038/nmeth.2019 PMC3855844

[nph70514-bib-0066] Schlüter U , Muschak M , Berger D , Altmann T . 2003. Photosynthetic performance of an Arabidopsis mutant with elevated stomatal density (sdd1‐1) under different light regimes. Journal of Experimental Botany 54: 867–874. 12554730 10.1093/jxb/erg087

[nph70514-bib-0067] Smith S , Weyers J , Berry W . 1989. Variation in stomatal characteristics over the lower surface of *Commelina communis* leaves. Plant, Cell & Environment 12: 653–659.

[nph70514-bib-0068] Sun J , Liu C , Hou J , He N . 2021. Spatial variation of stomatal morphological traits in grassland plants of the Loess Plateau. Ecological Indicators 128: 107857.

[nph70514-bib-0069] Tulva I , Koolmeister K , Hõrak H . 2024. Low relative air humidity and increased stomatal density independently hamper growth in young Arabidopsis. The Plant Journal 119: 2718–2736. 39072887 10.1111/tpj.16944

[nph70514-bib-0070] Vialet‐Chabrand S , Matthews JSA , Lawson T . 2021. Light, power, action! Interaction of respiratory energy‐ and blue light‐induced stomatal movements. New Phytologist 231: 2231–2246. 34101837 10.1111/nph.17538

[nph70514-bib-0071] Wall S , Lemonnier P , Milliken AL , Davey P , Lawson T . 2024. Simultaneous and independent abaxial and adaxial gas exchange measurements. In: Photosynthesis: methods and protocols. New York, NY, USA: Springer, 63–76. 10.1007/978-1-0716-3790-6_4 38649566

[nph70514-bib-0072] Wall S , Vialet‐Chabrand S , Davey P , Van Rie J , Galle A , Cockram J , Lawson T . 2022. Stomata on the abaxial and adaxial leaf surfaces contribute differently to leaf gas exchange and photosynthesis in wheat. New Phytologist 235: 1743–1756. 35586964 10.1111/nph.18257 PMC9545378

[nph70514-bib-0073] Watts JL , Dow GJ , Buckley TN , Muir CD . 2024. Does stomatal patterning in amphistomatous leaves minimize the CO_2_ diffusion path length within leaves? AoB Plants 16: plae015. 39906553 10.1093/aobpla/plae015 PMC11792893

[nph70514-bib-0074] Wei H , Jing Y , Zhang L , Kong D . 2021. Phytohormones and their crosstalk in regulating stomatal development and patterning. Journal of Experimental Botany 72: 2356–2370. 33512461 10.1093/jxb/erab034

[nph70514-bib-0075] Weyers J , Lawson T , Peng Z . 1997. Variation in stomatal characteristics at the whole‐leaf level. In: Scaling‐up: from cell to landscape. Cambridge University Press: Cambridge, UK.

[nph70514-bib-0083] Weyers JDB , Lawson T . 1997. Heterogeneity in stomatal characteristics. Advances in Botanical Research 26: 317–352. doi: 10.1016/S0065-2296(08)60124-X.

[nph70514-bib-0076] Wong S , Cowan I , Farquhar G . 1979. Stomatal conductance correlates with photosynthetic capacity. Nature 282: 424–426.

[nph70514-bib-0077] Xie J , Fernandes SB , Mayfield‐Jones D , Erice G , Choi ME , Lipka A , Leakey AD . 2021. Optical topometry and machine learning to rapidly phenotype stomatal patterning traits for maize QTL mapping. Plant Physiology 187: 1462–1480. 34618057 10.1093/plphys/kiab299 PMC8566313

[nph70514-bib-0078] Yasmeen S , Khan M , Khan I . 2020. Revisiting the physical mutagenesis for sugarcane improvement: a stomatal prospective. Scientific Reports 10: 412. 32994498 10.1038/s41598-020-73087-z PMC7524725

[nph70514-bib-0080] Zhen X , Zhang Y , López J , Qiu Y , Muehlbauer G , Sadok W . 2025. Leaf sheath stomata density is a driver of water use in a grass crop: genetic and physiological evidence on barley. Journal of Experimental Botany 45: 789. 10.1093/jxb/eraf067 39960857

[nph70514-bib-0081] Zuiderveld KJ . 1994. Contrast limited adaptive histogram equalization. Graphics Gems 4: 474–485.

