# Supplementary material for: Large‐scale quantification of stomatal patterning in barley leaves overexpressing epidermal patterning factor 1 reveals differential stomatal density between the adaxial and abaxial surfaces and spatial heterogeneity that impact stomatal function
Source: New Phytol. 2025 Aug 29;248(4):1769–83. doi: 10.1111/nph.70514 (PMC12529052; doi:10.1111/nph.70514)
Supplement: Supplementary file 2 — Fig. S1 Representative growth light spectrum measured at flag leaf level. Fig. S2 Leaf width variation between genotypes and positions along the barley leaf. Fig. S3 Manual validation of automated stomatal detection across 1 mm2 sampling areas. Table S1 Stomatal density and effect of EPF1 overexpression. Table S2 Coefficient of variation (%) in stomatal density. Table S3 Stomatal distribution ratios. Please note: Wiley is not responsible for the content or functionality of any Supporting Information supplied by the authors. Any queries (other than missing material) should be directed to the New Phytologist Central Office. [file NPH-248-1769-s002.pdf]

New Phytologist Supporting information.

Large scale quantification of stomatal patterning in Barley leaves over-expressing Epidermal patterning Factor 1 (EPF1) reveals differential stomatal density on the adaxial and abaxial surfaces and spatial heterogeneity which impacts stomatal function.

Mengjie Fan, Keri-Anne Moss, Pratham Jindal, Piotr Kasznicki, Philip Davey, Philippe P Laissue[*, #] and Tracy Lawson[1,#,*]

Article Acceptance date: 5th August 2025

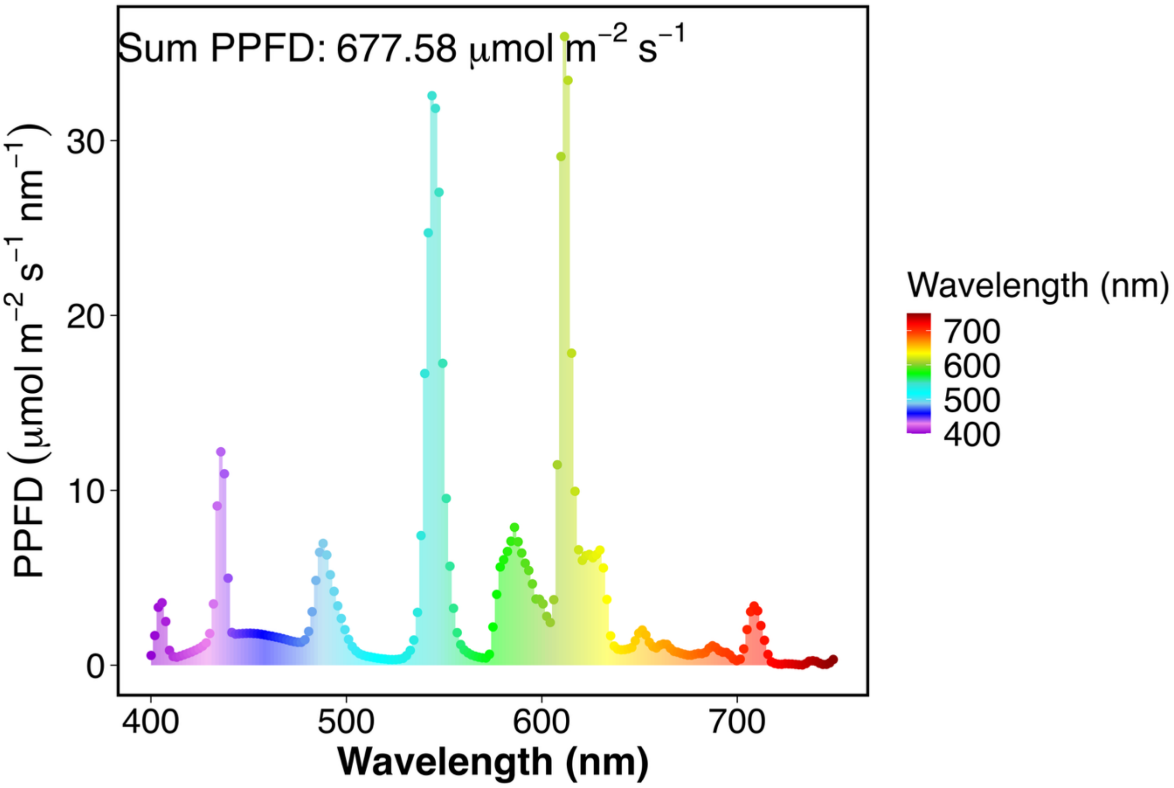

Supplementary Fig 1. Representative growth light spectrum measured at flag leaf level.

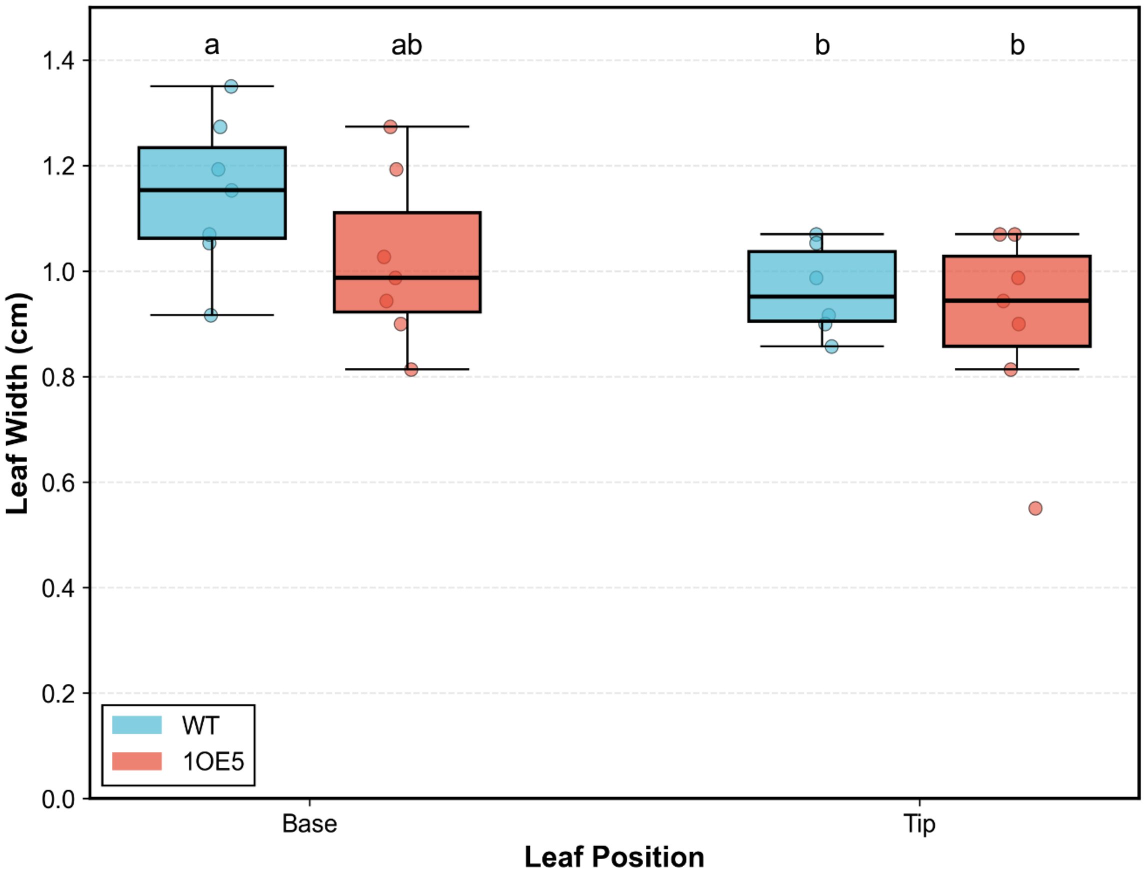

Supplementary Fig 2. Leaf width variation between genotypes and positions along the barley leaf. Different letters indicate significant differences between groups (Tukey HSD test, $p < 0.05$).

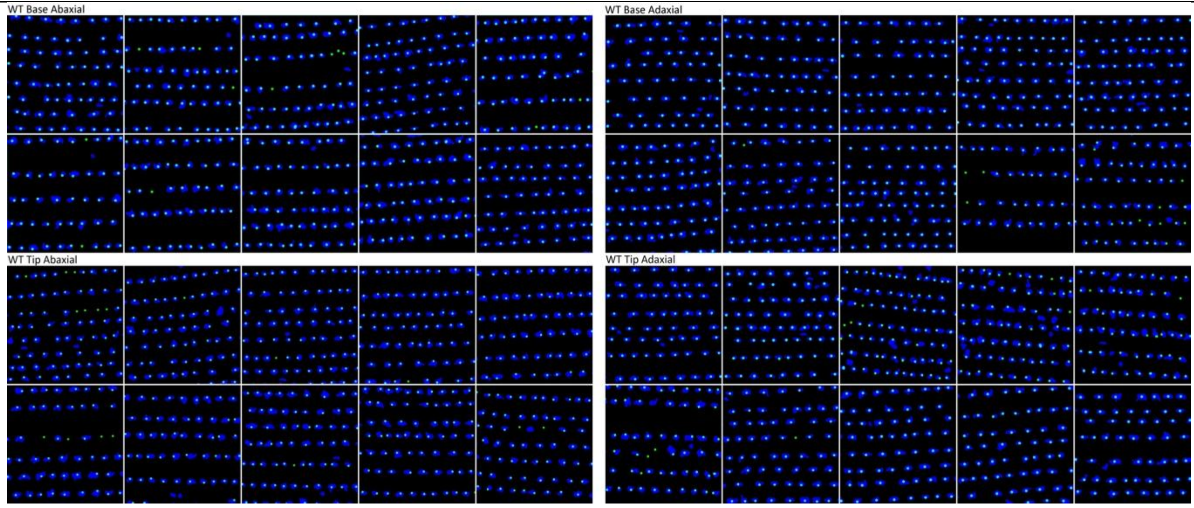

Supplementary Fig 3. Manual validation of automated stomatal detection across 1mm2 sampling areas. Ten randomly selected 1mm2 squares were analysed for each leaf region: Base abaxial, base adaxial, tip abaxial, and tip adaxial. Overall mean detection accuracy was 95.2% ± 3.6% across all leaf regions.

Supplementary Table 1. Stomatal density and effect of EPF1 overexpression

Location	WT	EPF1-OE	Reduction (%)	Effect Size (d)
Base Adaxial	58.93 ± 0.37^{f}	47.22 ± 0.33^{f}	19.9%	0.66
Base Abaxial	57.11 ± 0.47^{e}	23.14 ± 0.20^{e}	59.5%	2.01
Tip Adaxial	91.46 ± 0.43^{h}	54.75 ± 0.40^{h}	40.1%	1.85
Tip Abaxial	87.83 ± 0.56^{g}	30.87 ± 0.33^{g}	64.9%	2.75

Location	WT	EPF1-OE	Reduction (%)	Effect Size (d)

***Values represent mean ± SE (stomata/mm²).**

**Superscript letters indicate significant differences (Sidak, $p<0.05$)

Supplementary Table 2. **Coefficient of variation (%) in stomatal density**

Location	WT CV (%)	EPF1-OE CV (%)	CV Change
Base Adaxial	33.00908	32.63391	-1.1%
Base Abaxial	38.98110	46.18026	+18.5%
Tip Adaxial	23.55720	31.51703	+33.8%
Tip Abaxial	28.68949	46.21185	+61.1%

*CV Change shows percent increase/decrease in spatial heterogeneity in EPF1-OE

Supplementary Table 3. Stomatal distribution ratios

Ratio Type	Leaf Region	WT	1OE5
Adaxial:Abaxial	Base	1.03	2.04
Adaxial:Abaxial	Tip	1.04	1.77
Tip:Base	Adaxial	1.55	1.16
Tip:Base	Abaxial	1.54	1.33

*Values show relative stomatal density across leaf surfaces and positions
